# Supplementary material for: Glycoengineered keratinocyte library reveals essential functions of specific glycans for all stages of HSV-1 infection
Source: Nat Commun. 2023 Nov 2;14:7000. doi: 10.1038/s41467-023-42669-6 (PMC10622544; doi:10.1038/s41467-023-42669-6)

Glycoengineered keratinocyte library reveals essential functions of specific glycans for all stages of HSV-1 infection

Ieva Bagdonaite[1]*, Irina N Marinova[1], Asha M Rudjord-Levann[1], Emil MH Pallesen[1], Sarah King-Smith[1], Richard Karlsson[1], Troels B Rømer[1], Yen-Hsi Chen[1], Rebecca L Miller[1], Sigvard Olofsson[2], Rickard Nordén[2], Tomas Bergström[2], Sally Dabelsteen[3] and Hans H Wandall[1]*

[1] Copenhagen Center for Glycomics, Institute of Cellular and Molecular Medicine, University of Copenhagen, DK-2200 Copenhagen, Denmark

[2] Department of Infectious Diseases, Institute of Biomedicine, University of Gothenburg, SE-41346 Gothenburg, Sweden

[3] School of Dentistry, University of Copenhagen, DK-2200 Copenhagen, Denmark

Supplementary Information

Supplementary Table 1. Knock out indel sequencing and sgRNA/ZFN targeting sequences

Supplementary Table 2. CS disaccharide quantification in HaCaT WT

Supplementary Table 3. HS disaccharide quantification in HaCaT WT

Supplementary Table 4. Mutagenic primers

Supplementary Table 5. Oligonucleotide sequences for annealing and cloning the DNA template for gRNA into the LentiCRISPR-V2 plasmid

Supplementary Figure 1. N-glycoprofiling

Supplementary Figure 2. CORA O-glycoprofiling

Supplementary Figure 3. Flow cytometry supporting data

Supplementary Figure 4. Disaccharide analysis of HaCaT WT and *B4GALT7* KO cells

Supplementary Figure 5. Sequencing of DNA constructs

Supplementary Figure 6. Section panels of HSV-1-infected glycoengineered organotypic skin

Supplementary Table 1. Knock out indel sequencing and sgRNA/ZFN targeting sequences

Gene	Clone	Sequence[1]	gRNA/ZFN (5'-3')	Indel	First affected a.a.
N-Glycan					
MGAT1 KO	REF	GATtcgcctggcccaagacgccgAGG	TCGCCTGGCCCAAGACGCCG		
	1E7_A1	GATtcgcctggcccaag----cgCGG		-4	60
	1E7_A2	GATtcgcctggcccaagacg-cgAGG		-1	61
MGAT4A KO	REF	aatgagatgaggctccgcaaTGGAAC	AATGAGATGAGGCTCCGCAA		
	3C10_A1	aatgagatgaggctcc--aaTGGAAC		-2	4
	3C10_A2	aatgagatgaggctccgca-TGGAAC		-1	5
MGAT4B KO	REF	TTG…GCAggagagcctcaagc-gctccaAGGAGCTCAACCTGGTGCTGG	GGAGAGCCTCAAGCGCTCCA		
	3F8_A1	TTG…GCAggagagcctcaagcTgctccaAGGAGCTCAACCTGGTGCTGG		+1	60
	3F8_A2	TTG…--TGG		-96	36
MGAT5+4B KO	2B5 A1 (in *MGAT5* KO 1F12)	TTG…GCAgga--------------------GAGCTCAACCTGGTGCTGG		-19	56
MGAT5 KO	REF	GGGCCAtac-------gctggagtcatgacagcTTAT	GCTGTCATGACTCCAGCGTA		
	1F12_A1	GGGCCAtacA------gctggagtcatgacagcTTAT		+1	74
	1F12_A2	GGGCCACATGACATAGgctggagtcatgacagcTTAT		+7	73
O-GalNAc					
C1GALT1C1 KO[2]	REF	ATCACCCCAACCAGGTAGTagaaggctGTTGTTCAGATAT	AGAAGGCT (ZFN cut site)		
	D5_A1	ATCACCCCAACCAGGTA----------GTTGTTCAGATAT		-10	267

[1] Sequences are shown in the orientation of the gene, with sgRNA recognition sequences (direct or complementary)/ZFN cut sites in lower case and PAM sequences underlined

[2] Radhakrishnan *et al.*, 2014 (DOI: 10.1073/pnas.1406619111)

C1GALT1 KO	REF	GCCCAgcgttgtaacaaagtgttgtTTATGAGTTCAGAA	ACAACACTTTGTTACAACGC		
	2E5_A1	GCCCAG--TTGTAACAAAGTGTTGTTTATGAGTTCAGAA		-2	114
	2E5_A2	GCCCAGCG-----------TGTTGTTTATGAGTTCAGAA		-11	115
GCNT1 KO	REF	AATCACCTtctccgttttaaggattcatCAA	ATGAATCCTTAAAACGGAGA		
	2E7_A1	AAT-------------ttaaggattcatCAA		-13	27
	2E7_A2	AATCACCTtc--cgttAATGggattcatCAA		-2	30
ST3GAL1 KO	REF	TGGTGCCCttc-aagaccatcgacttggaGTGGG	TCCAAGTCGATGGTCTTGAA		
	2B2_A1	TGGTGCCCttcAaagaccatcgacttggaGTGGG		+1	210
GAG					
B4GALT7 KO	REF	TGCACGACGTtgacctgctccctctcaa-cgAGGAGCTGGAC	TGACCTGCTCCCTCTCAACG		
	3D9_A1	TGCACGACGTtgacctgctccctctcaaAcgAGGAGCTGGAC		+1	170
	3D9_A2	TGCACGACGTtgacctgctccctctc--------------AC		-13	170
GSL					
B4GALT5 KO	REF	AGCCTTCTGATTGCat-------------gccTCGGTGGAAG	ATGCC (ZFN cut site)		
	D11_A1	AGCCTTCTGATTGCATGTAGAATTCCCTAGCCTCGGTGGAAG		+13	
					160
ST3GAL5 KO	REF	GTTatttgagcacaggtata-gcgTGGACTTAC	ATTTGAGCACAGGTATAGCG		
	1C5_A1	GTTatttgagcacaggtataCgcgTGGACTTAC		+1	138
	1C5_A2	GTTatttgagcGTAA---------TGGACTTAC		-8	135

Supplementary Table 2. CS disaccharide quantification in HaCaT WT.

Standards						HaCaT WT			
Name	MW	Abbreviation	Elution time, min	Fluorescence signal intensity	Picomoles/ fluoresc. unit	Fluorescence signal intensity	Picomoles	Molar %	µg
UA2S-GalNAc4S	605.388	D2A4	24.060	2279265	0.000008775	1102217	9.6717	1.70	0.006
UA-GalNAc4S6S	605.388	D0A10	26.241	2525125	0.000007920	8385507	66.4166	11.70	0.040
UA2S-GalNAc6S	605.388	D2A6	26.754	3868465	0.000005170	1732606	8.9576	1.58	0.005
UA-GalNAc4S	503.344	D0A4	29.134	1997232	0.000010014	32580632	326.2579	57.46	0.164
UA2S-GalNAc	503.344	D2A0	29.412	5229740	0.000003824	0	0.0000	0.00	0.000
UA-GalNAc6S	503.344	D0A6	30.810	2541577	0.000007869	18234958	143.4933	25.27	0.072
UA-GalNAc	401.300	D0A0	33.046	2631878	0.000007599	1706858	12.9706	2.28	0.005

Supplementary Table 3. HS disaccharide quantification in HaCaT WT.

Standards						HaCaT WT			
Name	MW	Abbreviation	Elution time, min	Fluorescence signal intensity	Picomoles/ fluoresc. unit	Fluorescence signal intensity	Picomoles	Molar %	µg
UA-GlcNAc	401.300	IV-A	52.718	1509711	0.00001325	12516037	165.8071	15.29	0.067
UA2S-GlcNAc	503.344	III-A	47.949	1930891	0.00001036	2358398	24.4281	2.25	0.012
UA-GlcNAc6S	503.344	II-A	40.829	1923938	0.00001040	ND*	0.0000	0.00	0.000
UA2S-GlcNAc6S	605.388	I-A	37.379	1599135	0.00001251	0	0.0000	0.00	0.000
UA-GlcNH$_2$	337.281	IV-H	37.996	2105780	0.00000950	0	0.0000	0.00	0.000
UA2S-GlcNH$_2$	461.307	III-H	35.017	1596156	0.00001253	59749	0.7487	0.07	0.000
UA-GlcNH$_2$6S	461.307	II-H	30.481	1591894	0.00001256	0	0.0000	0.00	0.000
UA2S-GlcNH$_2$6S	564.359	I-H	28.037	1643712	0.00001217	0	0.0000	0.00	0.000
UA-GlcNS	461.307	IV-S	25.568	1710782	0.00001169	32235845	376.8551	34.75	0.174
UA2S-GlcNS	563.351	III-S	20.983	1578920	0.00001267	22187109	281.0416	25.91	0.158
UA-GlcNS6S	563.351	II-S	16.089	1254200	0.00001595	4449247	70.9496	6.54	0.040
UA2S-GlcNS6S	665.395	I-S	13.790	1904574	0.00001050	15293468	160.5973	14.81	0.107
UA-GlcNS3S	563.351	VIII-S	14.708	1645590	0.00001215	64732	0.7867	0.07	0.000
UA2S-GlcNS3S	665.395	VII-S	14.213	1936850	0.00001033	ND*	0.0000	0.00	0.000
UA-GlcNS3S6S	665.395	VI-S	10.962	1452743	0.00001377	25165	0.3464	0.03	0.000
UA2S-GlcNS3S6S	767.439	V-S	10.459	1428129	0.00001400	209107	2.9284	0.27	0.002

*ND – not quantified due to overlap with other peaks

Supplementary Table 4. Sequences of mutagenic primers. Altered nucleotides are shown in red.

Name	5’ to 3’	Length
gB_T169A	GTACTACAAAGACGTCGCCGTTTCGCAGGTGTG	33
gB_T267A	CCACCGGTACGGGGCGACGGTAAACTG	27
gB_T268A	ACCGGTACGGGACGGCGGTAAACTGCATC	29
gB_T267A_T268A	TCCACCGGTACGGGGCGGCGGTAAACTGCATC	32
gB_T690A	CCTGGAGGTGTACGCCCGCCACGAGATC	28
gB_T703A	CCTGCTGGACTACGCGGAGGTCCAGCG	27
gD_S33A	CCTTGGCGGATGCCGCTCTCAAGATGGCC	29
gD_T255A	CGAGAACCAGCGCGCCGTCGCCGTATA	27
gD_S260A	ACCGTCGCCGTATACGCCTTGAAGATCGCCGG	32
gD_T255A_S260A	CGAGAACCAGCGCGCCGTCGCCGTATACGCCTTGAAGATCGCCGG	45

Supplementary Table 5. Oligonucleotide sequences for annealing and cloning the DNA template for gRNA into the LentiCRISPR-V2 plasmid.

Target gene	gRNA targeting sequence (5' to 3')	5' oligonucleotide sequence (5' to 3')	3' oligonucleotide sequence (5' to 3')
MGAT1	TCGCCTGGCCCAAGACGCCG	CACCGTCGCCTGGCCCAAGACGCCG	AAACCGGCGTCTTGGGCCAGGCGAC
MGAT4A	AATGAGATGAGGCTCCGCAA	CACCGAATGAGATGAGGCTCCGCAA	AAACTTGCGGAGCCTCATCTCATTC
MGAT4B	GGAGAGCCTCAAGCGCTCCA	CACCGGGAGAGCCTCAAGCGCTCCA	AAACTGGAGCGCTTGAGGCTCTCCC
MGAT5	GCTGTCATGACTCCAGCGTA	CACCGGCTGTCATGACTCCAGCGTA	AAACTACGCTGGAGTCATGACAGCC
C1GALT1	ACAACACTTTGTTACAACGC	CACCGACAACACTTTGTTACAACGC	AAACGCGTTGTAACAAAGTGTTGTC
GCNT1	ATGAATCCTTAAAACGGAGA	CACCGATGAATCCTTAAAACGGAGA	AAACTCTCCGTTTTAAGGATTCATC
ST3GAL1	TCCAAGTCGATGGTCTTGAA	CACCGTCCAAGTCGATGGTCTTGAA	AAACTTCAAGACCATCGACTTGGAC
B4GALT7	TGACCTGCTCCCTCTCAACG	CACCGTGACCTGCTCCCTCTCAACG	AAACCGTTGAGAGGGAGCAGGTCAC
ST3GAL5	ATTTGAGCACAGGTATAGCG	CACCGATTTGAGCACAGGTATAGCG	AAACCGCTATACCTGTGCTCAAATC

Supplementary Figure 1. N-glycoprofling

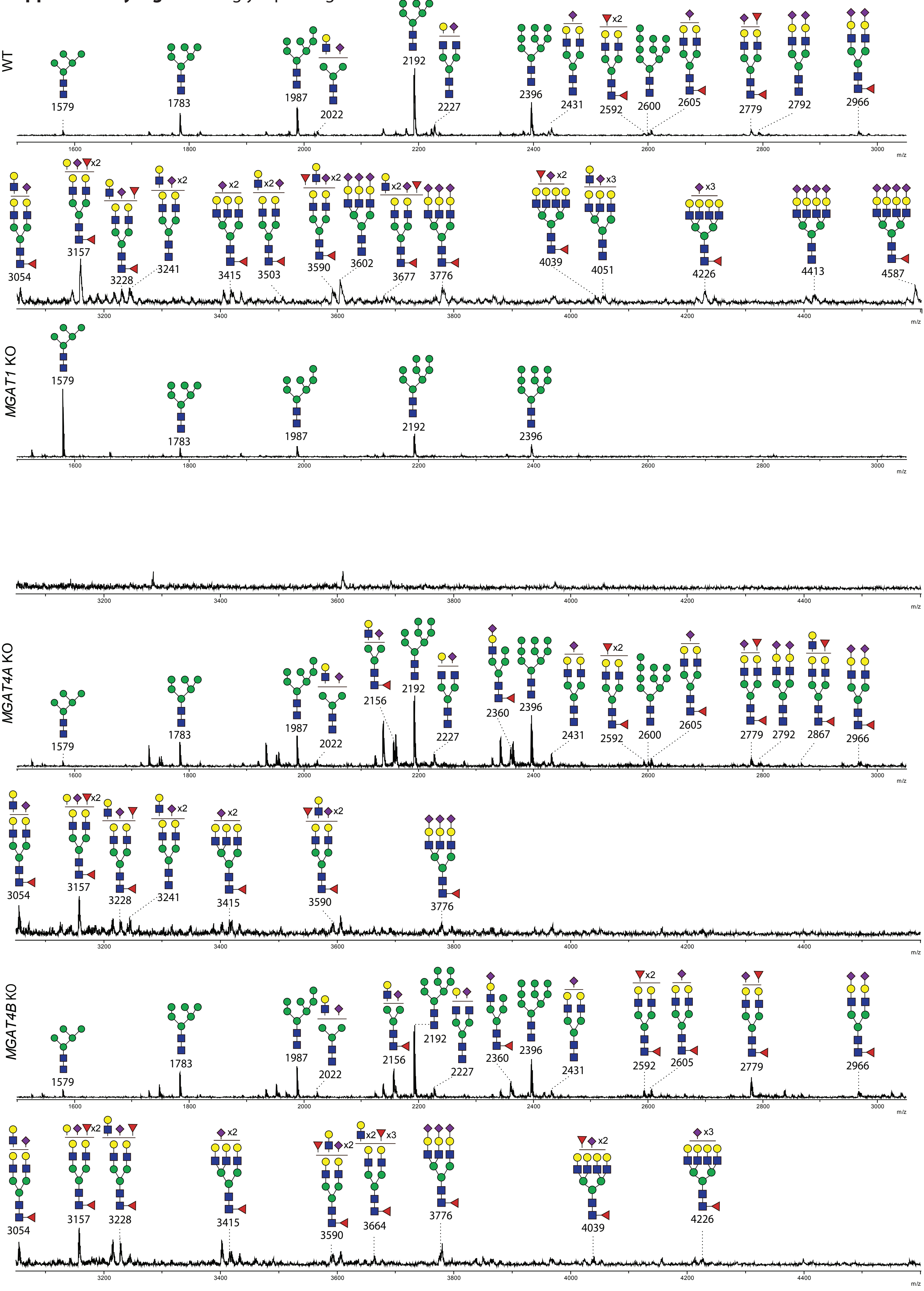

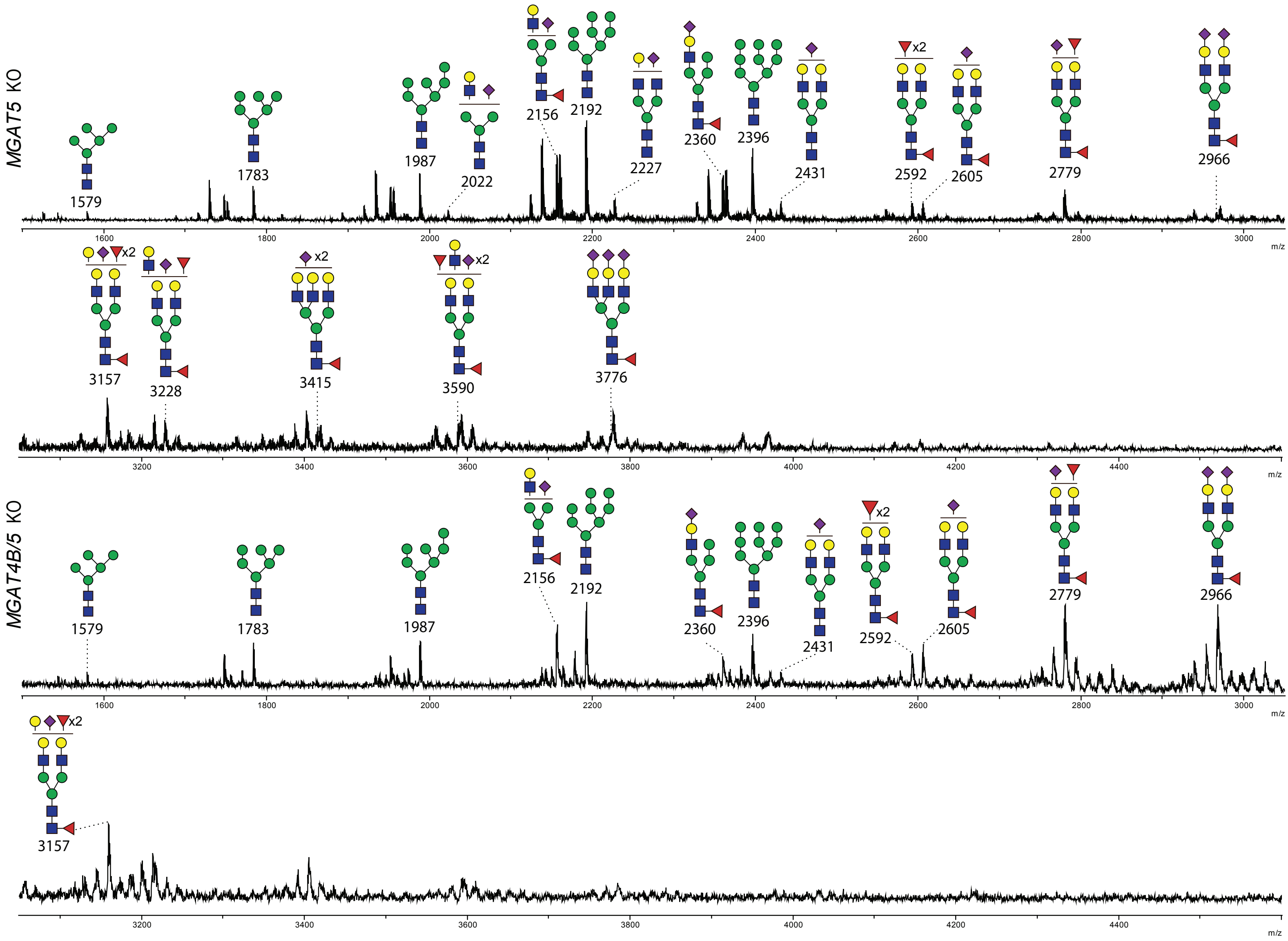
MGAT5 KO
1579
1783
1987
2022
2156
2192
2227
2360
2396
2431
x2
2592
2605
2779
2966
1600 1800 2000 2200 2400 2600 2800 3000
m/z
x2
3157
3228
x2
3415
x2
3590
3776
3200 3400 3600 3800 4000 4200 4400
m/z
MGAT4B/5 KO
1579
1783
1987
2156
2192
2360
2396
2431
x2
2592
2605
2779
2966
1600 1800 2000 2200 2400 2600 2800 3000
m/z
x2
3157
3200 3400 3600 3800 4000 4200 4400
m/z

Supplementary Figure 2. CORA O-glycoprofling

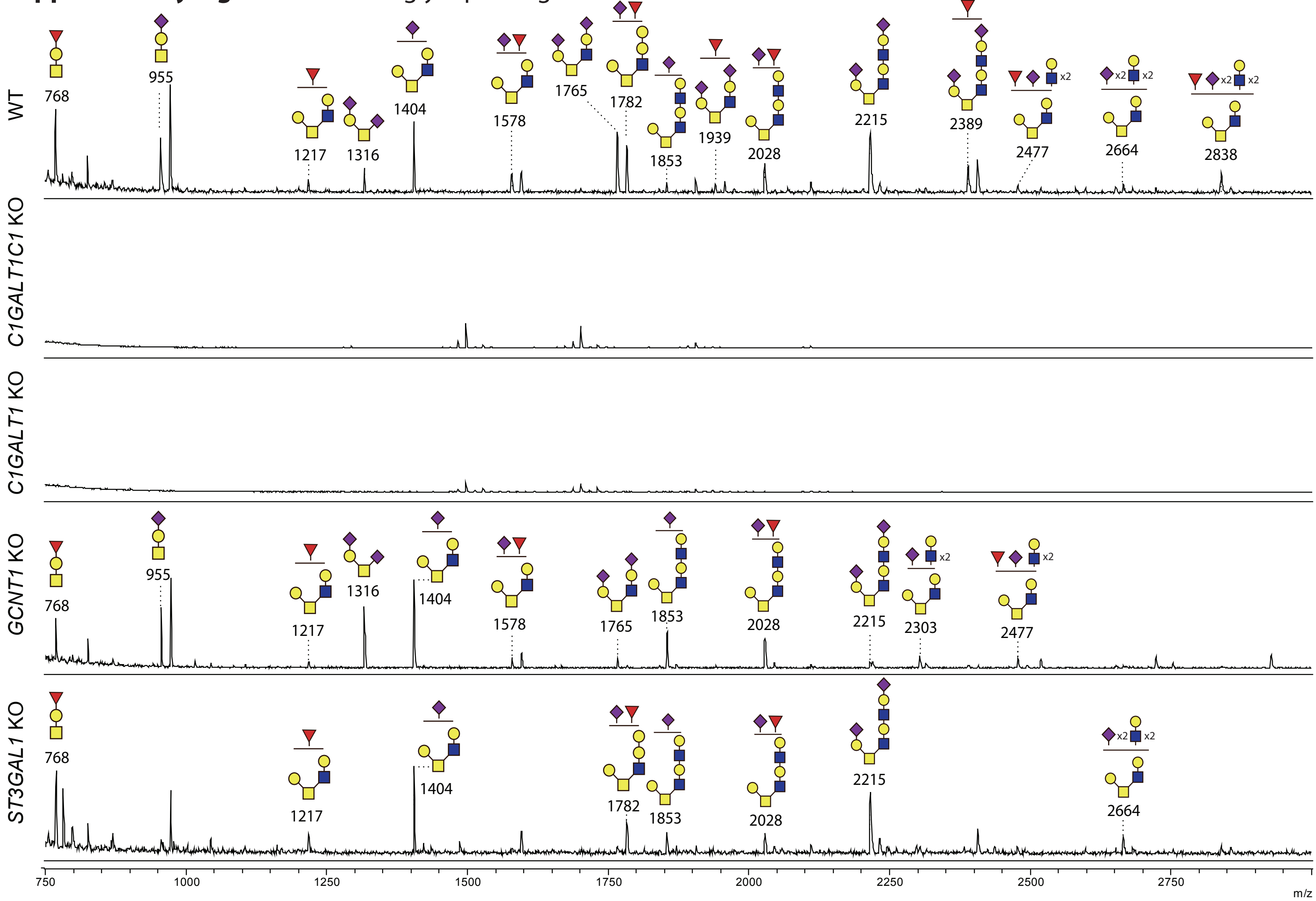

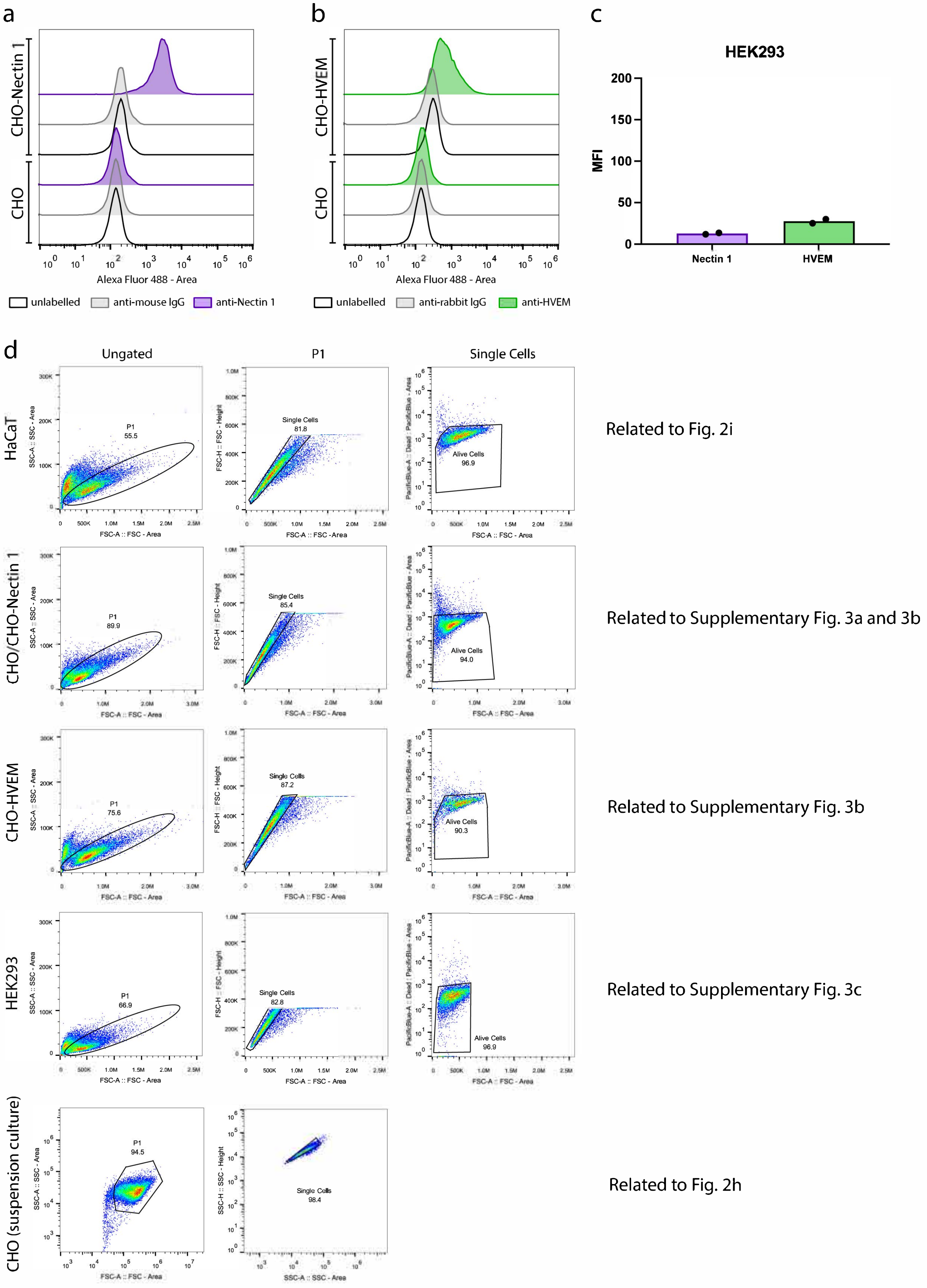

Supplementary Figure 3. Flow cytometry supporting data. a Nectin 1 cell surface expression in CHO-Nectin 1 and parental CHO cell line. **b** HVEM cell surface expression in CHO-HVEM and parental CHO cell line. **c** Nectin 1 and HVEM cell surface expression in HEK293 cells. Background corrected median fluorescence intensity from two independent experiments is shown. **d** Gating strategies for various cell lines used for flow cytometry.

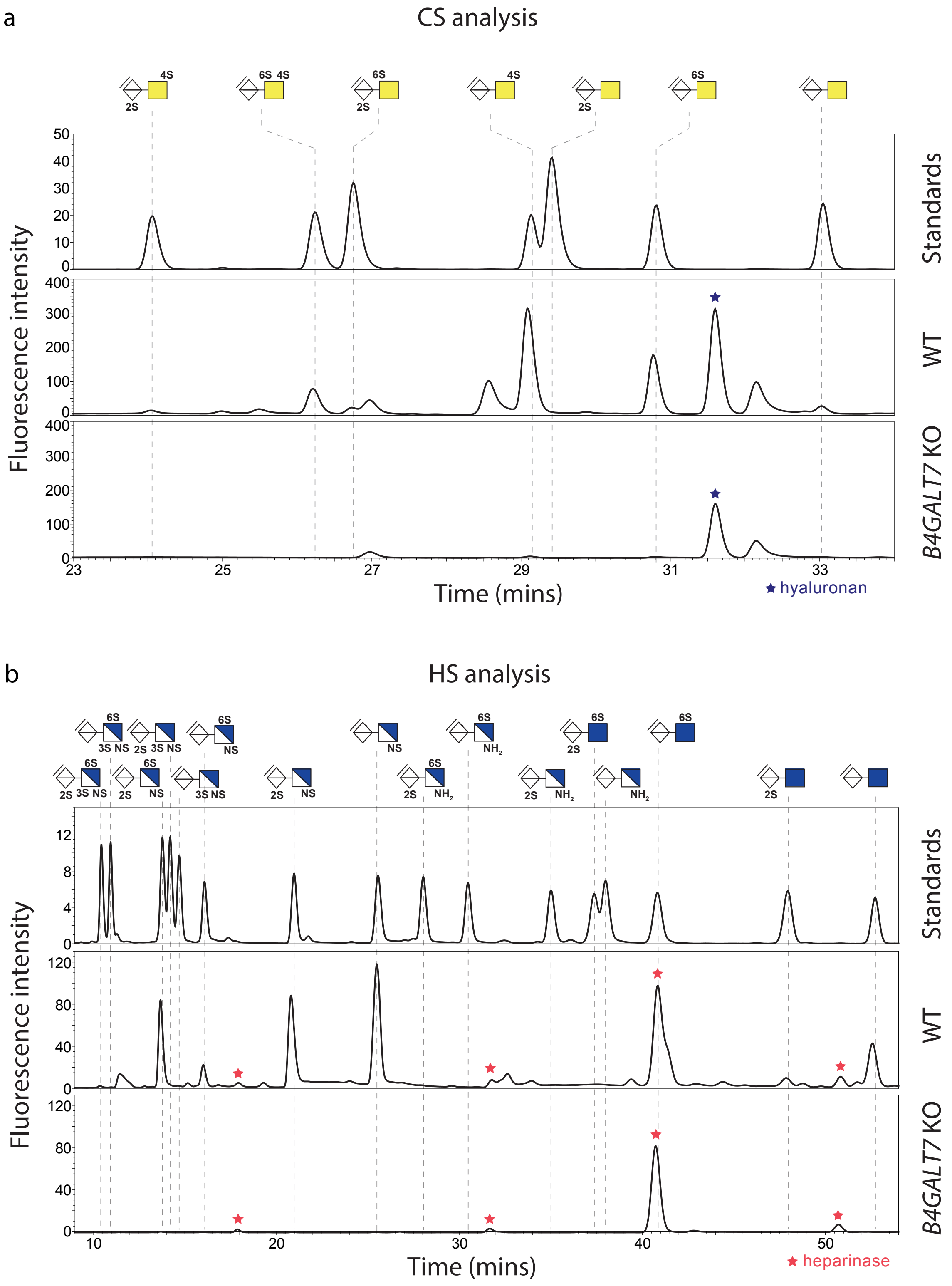

Supplementary Figure 4. Disaccharide analysis of HaCaT WT and *B4GALT7* KO cells. a Chondroitin sulfate (CS) analysis. **b** Heparan sulfate (HS) analysis.

Supplementary Figure 5. Sequencing of DNA constructs. Multiple sequence alignment for the DNA regions corresponding to the positions of introduced single amino acid mutations in HSV-1 gB and gD expression constructs. Asterisks indicate identical nucleotides.

```
gB Tyr165-Val173          TyrLysAspValThrValSerGlnVal
                           Y  K  D  V  T  V  S  Q  V
gB_WT                     TACAAAGACGTCACCGTTTCGCAGGTG   27
gB_T169A                  TACAAAGACGTCGCCGTTTCGCAGGTG   27
gB_T267A                  TACAAAGACGTCACCGTTTCGCAGGTG   27
gB_T268A                  TACAAAGACGTCACCGTTTCGCAGGTG   27
gB_T267A_T268A            TACAAAGACGTCACCGTTTCGCAGGTG   27
gB_T690A                  TACAAAGACGTCACCGTTTCGCAGGTG   27
gB_T703A                  TACAAAGACGTCACCGTTTCGCAGGTG   27
gB_T690A_T703A            TACAAAGACGTCACCGTTTCGCAGGTG   27
gB_T169A_T267A_T268A      TACAAAGACGTCGCCGTTTCGCAGGTG   27
                          ************ **************

gB His263-Ile272          HisArgTyrGlyThrThrValAsnCysIle
                           H  R  Y  G  T  T  V  N  C  I
gB_WT                     CACCGGTACGGGACGACGGTAAACTGCATC   30
gB_T169A                  CACCGGTACGGGACGACGGTAAACTGCATC   30
gB_T267A                  CACCGGTACGGGGCGACGGTAAACTGCATC   30
gB_T268A                  CACCGGTACGGGACGGCGGTAAACTGCATC   30
gB_T267A_T268A            CACCGGTACGGGGCGGCGGTAAACTGCATC   30
gB_T690A                  CACCGGTACGGGACGACGGTAAACTGCATC   30
gB_T703A                  CACCGGTACGGGACGACGGTAAACTGCATC   30
gB_T690A_T703A            CACCGGTACGGGACGACGGTAAACTGCATC   30
gB_T169A_T267A_T268A      CACCGGTACGGGGCGGCGGTAAACTGCATC   30
                          ************ ** **************

gB Glu687-Gln706          GluValTyrThrArgHisGluIleLysAspSerGlyLeuLeuAspTyrThrGluValGln
                           E  V  Y  T  R  H  E  I  K  D  S  G  L  L  D  Y  T  E  V  Q
gB_WT                     GAGGTGTACACCCGCCACGAGATCAAGGACAGCGGCCTGCTGGACTACACGGAGGTCCAG   60
gB_T169A                  GAGGTGTACACCCGCCACGAGATCAAGGACAGCGGCCTGCTGGACTACACGGAGGTCCAG   60
gB_T267A                  GAGGTGTACACCCGCCACGAGATCAAGGACAGCGGCCTGCTGGACTACACGGAGGTCCAG   60
gB_T268A                  GAGGTGTACACCCGCCACGAGATCAAGGACAGCGGCCTGCTGGACTACACGGAGGTCCAG   60
gB_T267A_T268A            GAGGTGTACACCCGCCACGAGATCAAGGACAGCGGCCTGCTGGACTACACGGAGGTCCAG   60
gB_T690A                  GAGGTGTACGCCCGCCACGAGATCAAGGACAGCGGCCTGCTGGACTACACGGAGGTCCAG   60
gB_T703A                  GAGGTGTACACCCGCCACGAGATCAAGGACAGCGGCCTGCTGGACTACGCGGAGGTCCAG   60
gB_T690A_T703A            GAGGTGTACGCCCGCCACGAGATCAAGGACAGCGGCCTGCTGGACTACGCGGAGGTCCAG   60
gB_T169A_T267A_T268A      GAGGTGTACACCCGCCACGAGATCAAGGACAGCGGCCTGCTGGACTACACGGAGGTCCAG   60
                          ********* ************************************** ***********

gD Leu29-Ala37            LeuAlaAspAlaSerLeuLysMetAla
                           L  A  D  A  S  L  K  M  A
gD_WT                     TTGGCGGATGCCTCTCTCAAGATGGCC   27
gD_S33A                   TTGGCGGATGCCGCTCTCAAGATGGCC   27
gD_T255A                  TTGGCGGATGCCTCTCTCAAGATGGCC   27
gD_S260A                  TTGGCGGATGCCTCTCTCAAGATGGCC   27
gD_T255A_S260A            TTGGCGGATGCCTCTCTCAAGATGGCC   27
gD_S33A_T255A_S260A       TTGGCGGATGCCGCTCTCAAGATGGCC   27
                          ************ **************

gD Glu251-Ala264          GluAsnGlnArgThrValAlaValTyrSerLeuLysIleAla
                           E  N  Q  R  T  V  A  V  Y  S  L  K  I  A
gD_WT                     GAGAACCAGCGCACCGTCGCCGTATACAGCTTGAAGATCGCC   42
gD_S33A                   GAGAACCAGCGCACCGTCGCCGTATACAGCTTGAAGATCGCC   42
gD_T255A                  GAGAACCAGCGCGCCGTCGCCGTATACAGCTTGAAGATCGCC   42
gD_S260A                  GAGAACCAGCGCACCGTCGCCGTATACGCCTTGAAGATCGCC   42
gD_T255A_S260A            GAGAACCAGCGCGCCGTCGCCGTATACGCCTTGAAGATCGCC   42
gD_S33A_T255A_S260A       GAGAACCAGCGCGCCGTCGCCGTATACGCCTTGAAGATCGCC   42
                          ************ ************** **************
```

Supplementary Figure 6. Section panels of HSV-1-infected glycoengineered organotypic skin. 36 hours post-infection the tissues were fixed in formalin and two independent series of 10 consecutive sections with 30 µm intervals were stained with goat anti-HSV-1 FITC pAb followed by imaging with a fluorescence-equipped slide scanner.

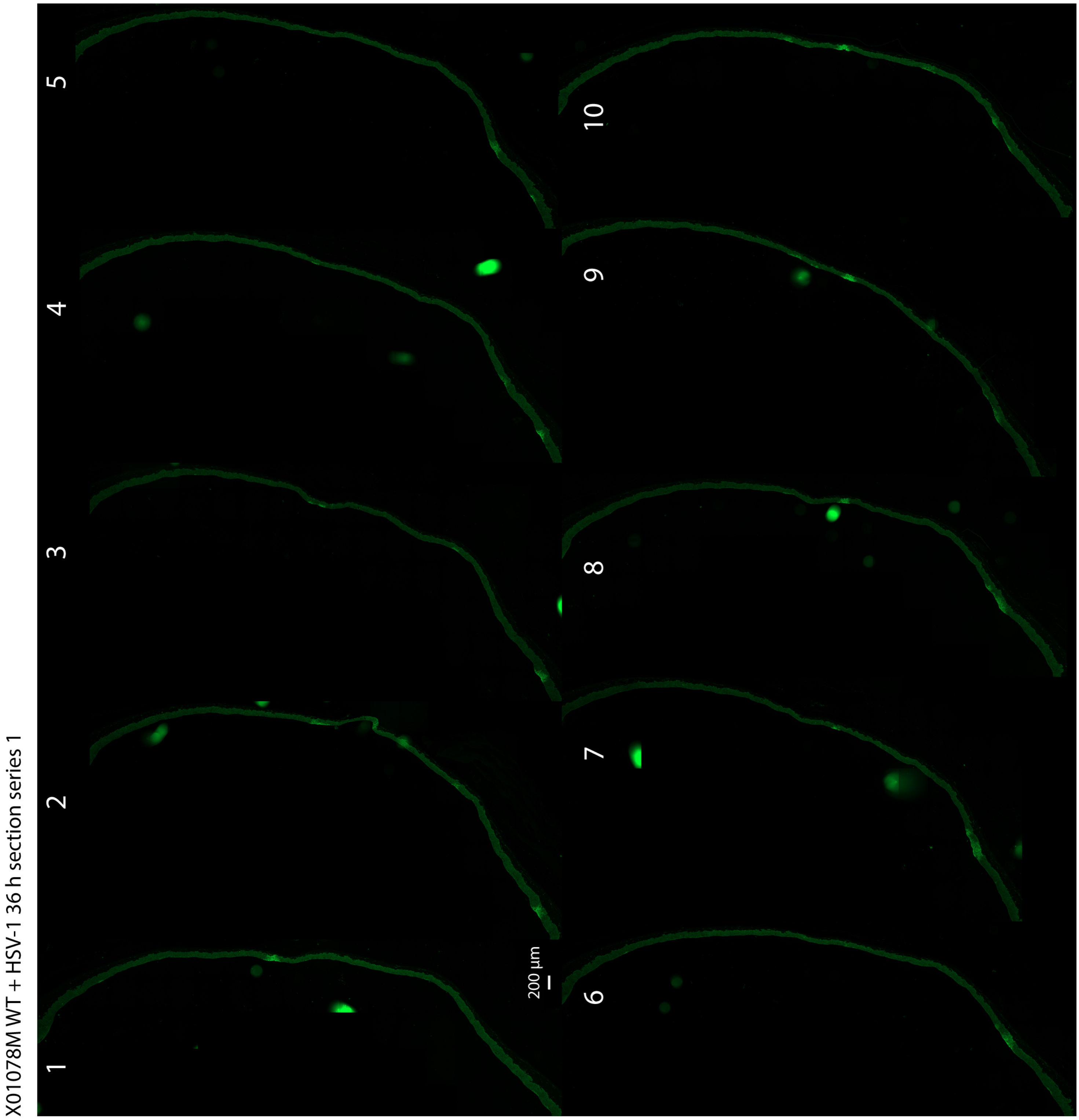

X01078M WT + HSV-1 36 h section series 2

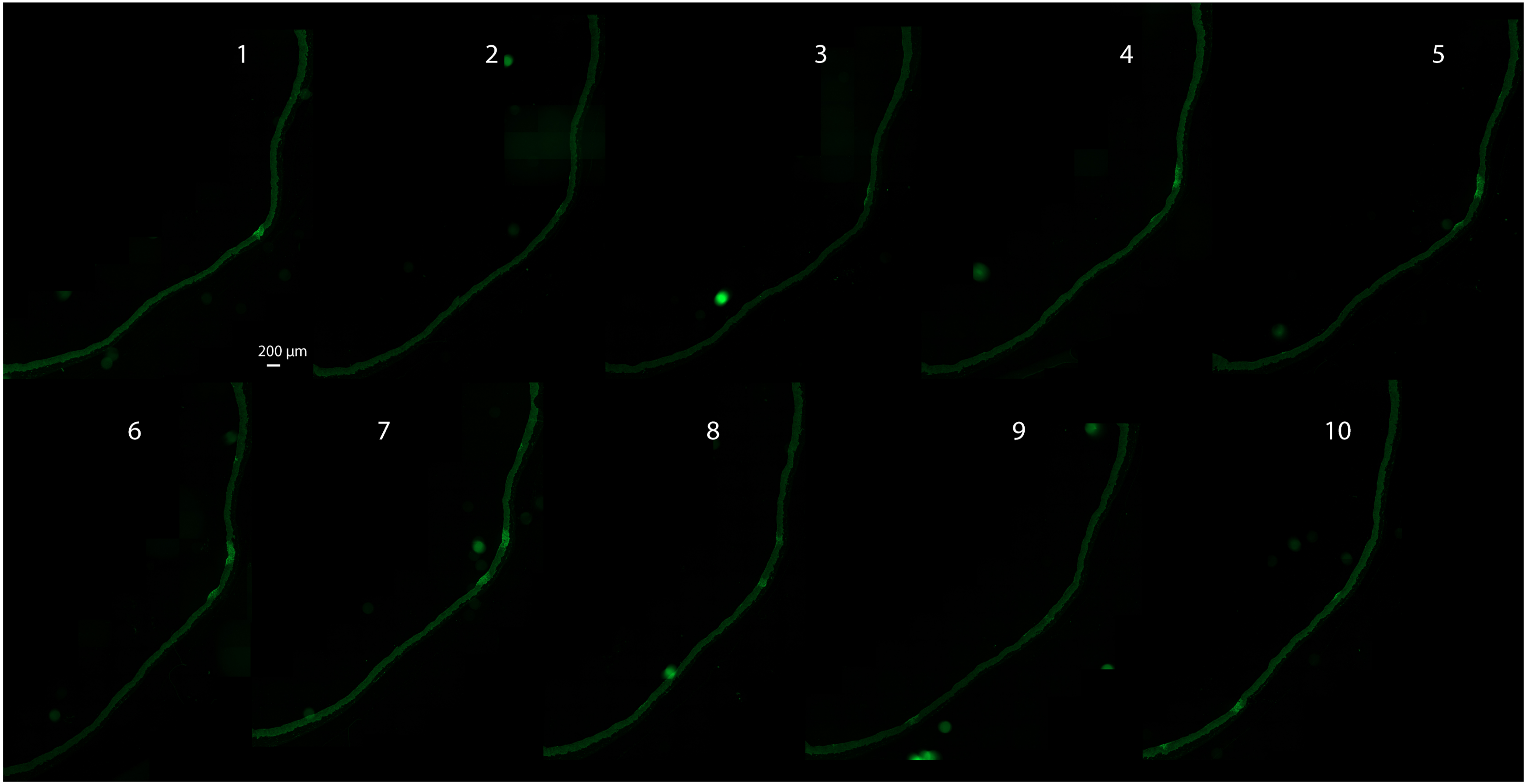

X01078O *MGAT1* KO 1E7 + HSV-1 36 h section series 1

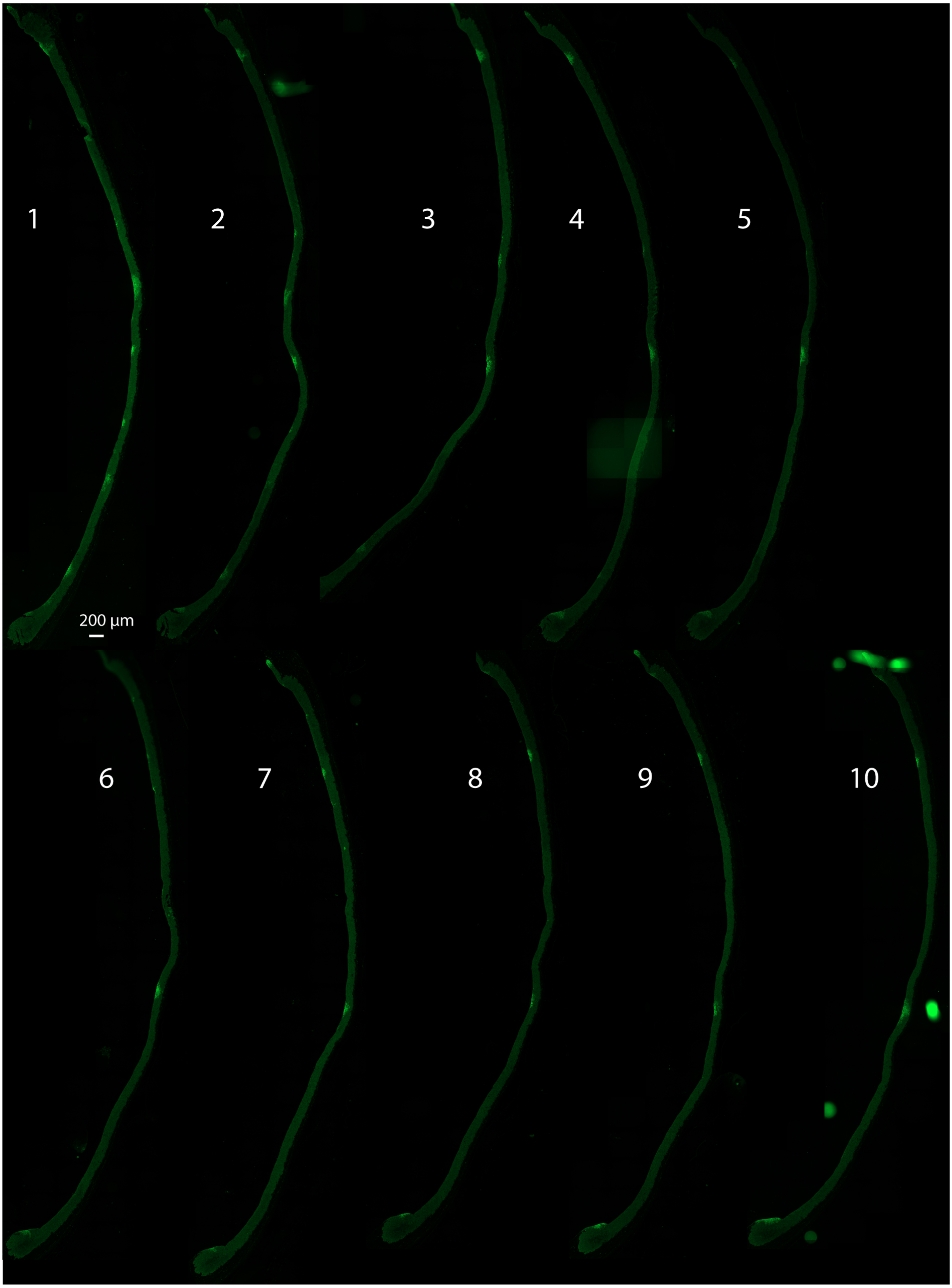

X01078O *MGAT1* KO 1E7 + HSV-1 36 h section series 2

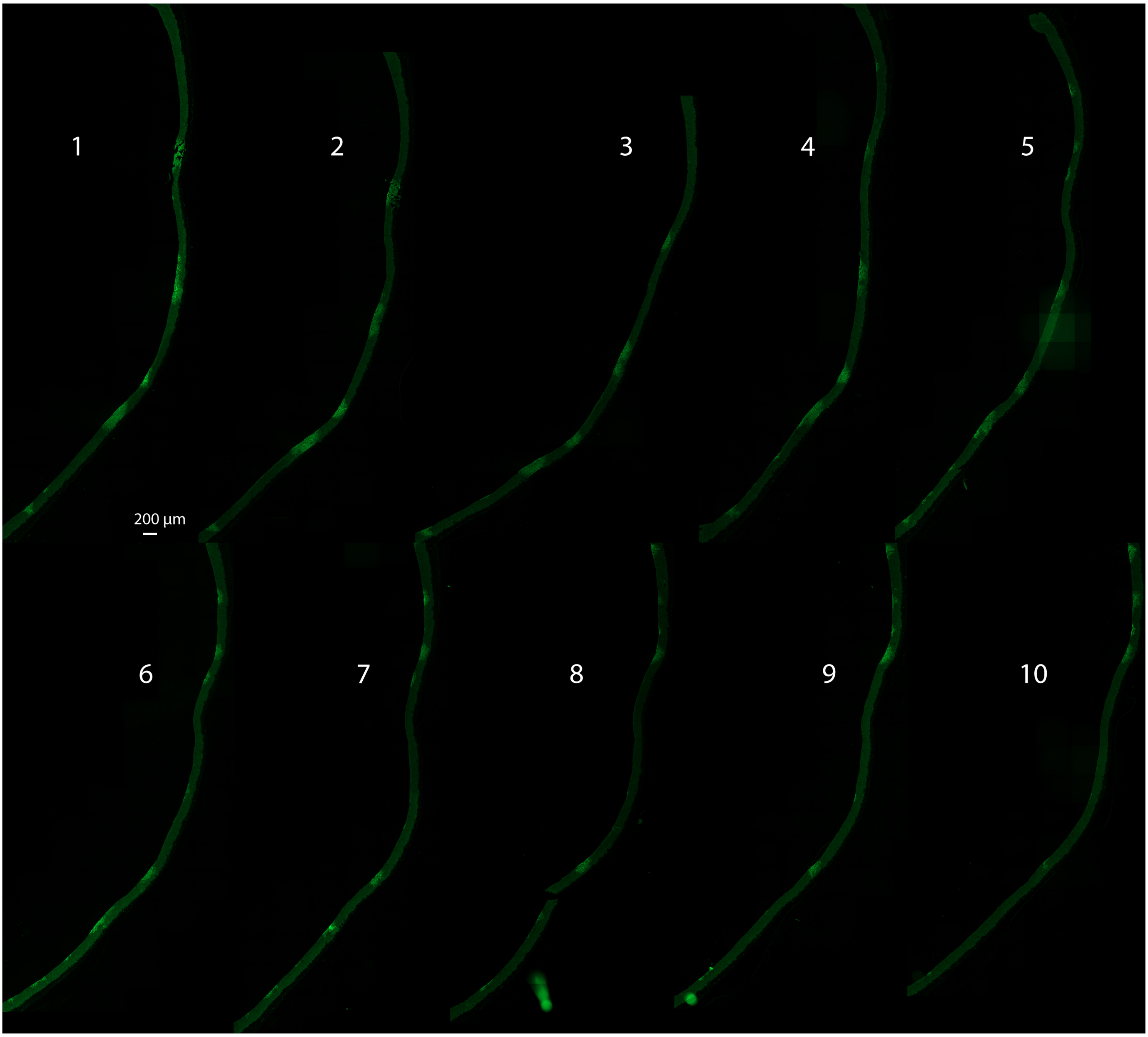

X01078P *MGAT4A* KO 3C10 + HSV-1 36 h section series 1

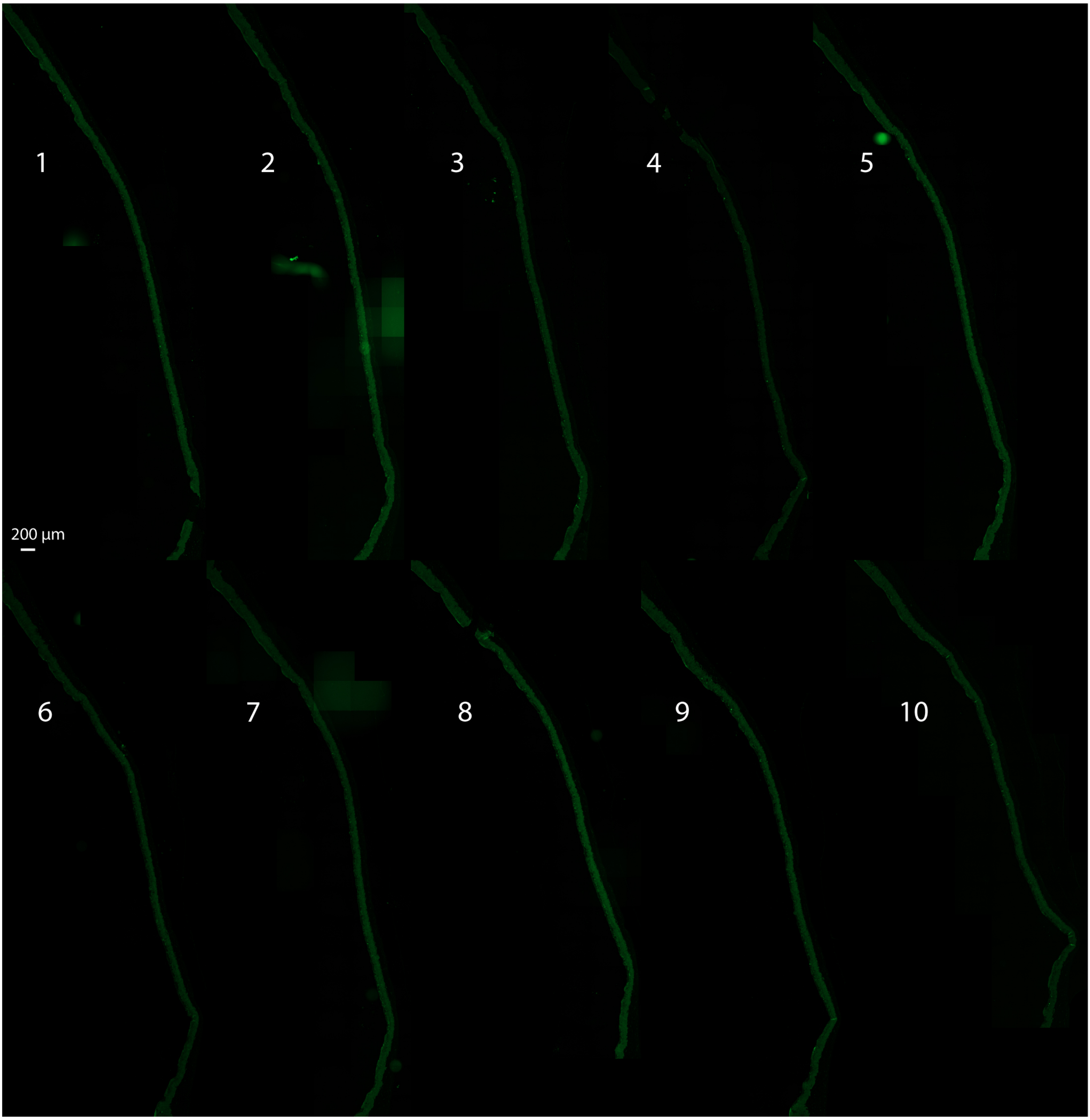

X01078P *MGAT4A* KO 3C10 + HSV-1 36 h section series 2

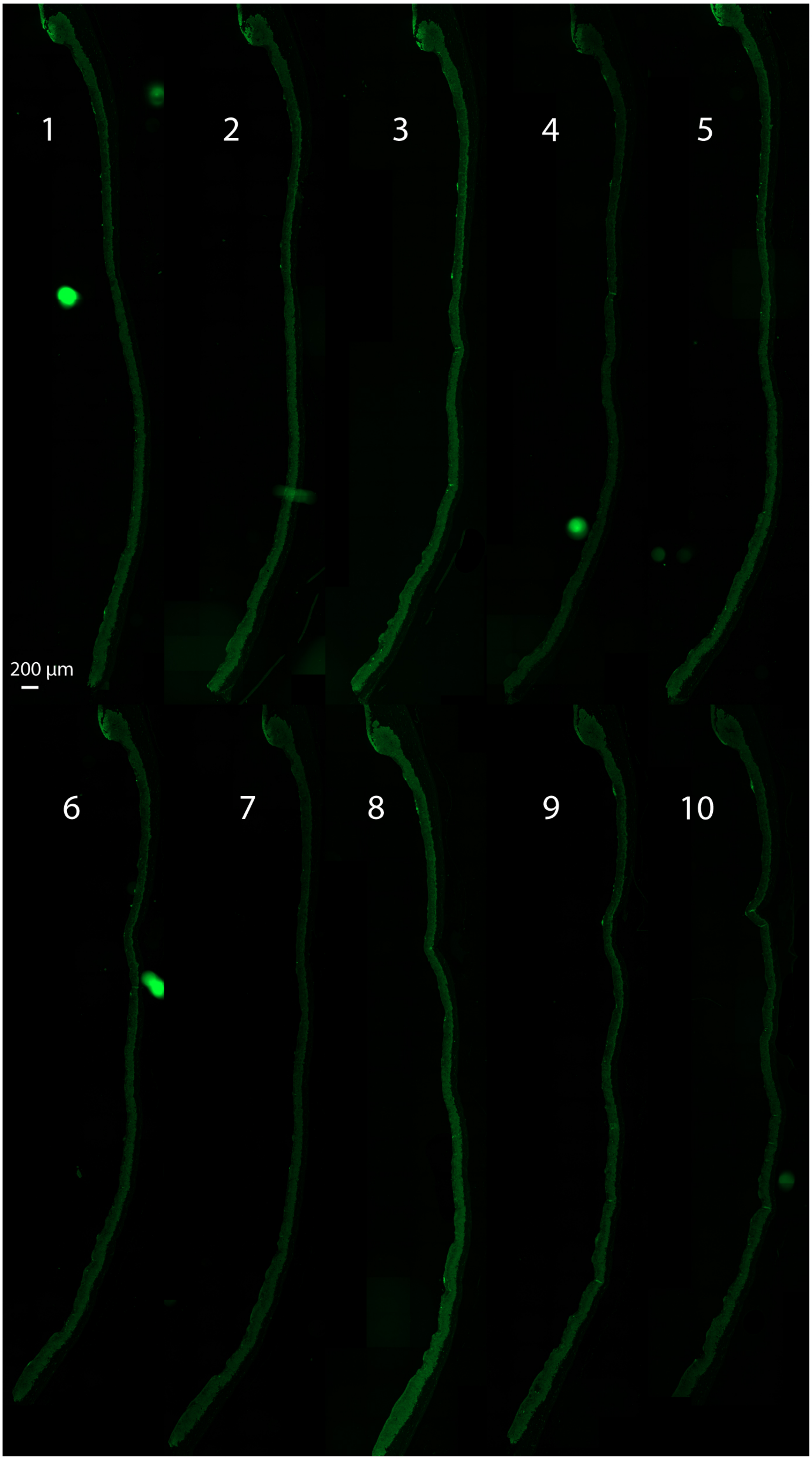

X01105L *MGAT4B* KO 3F8 + HSV-1 36 h section series 1

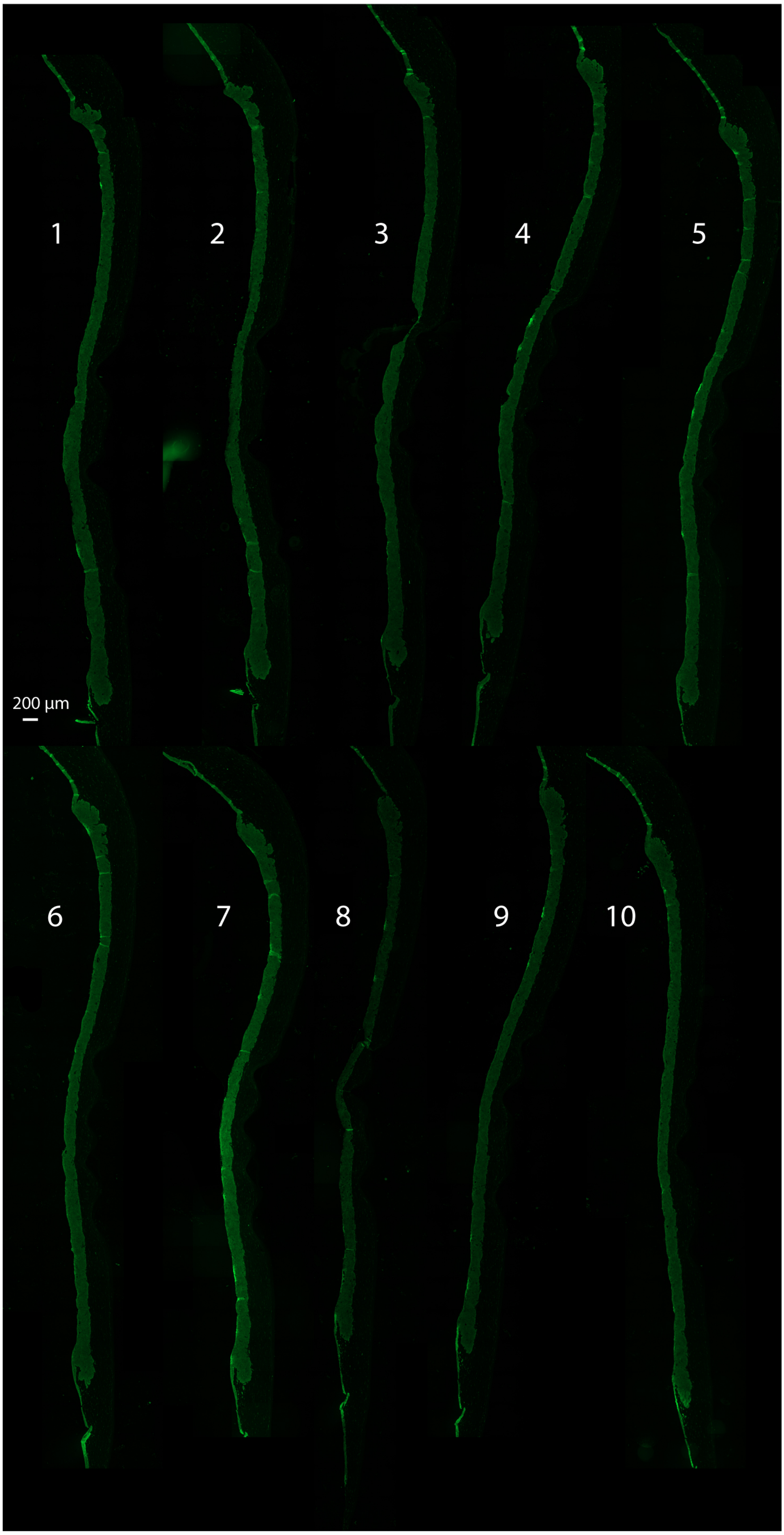

X01105L *MGAT4B* KO 3F8 + HSV-1 36 h section series 2

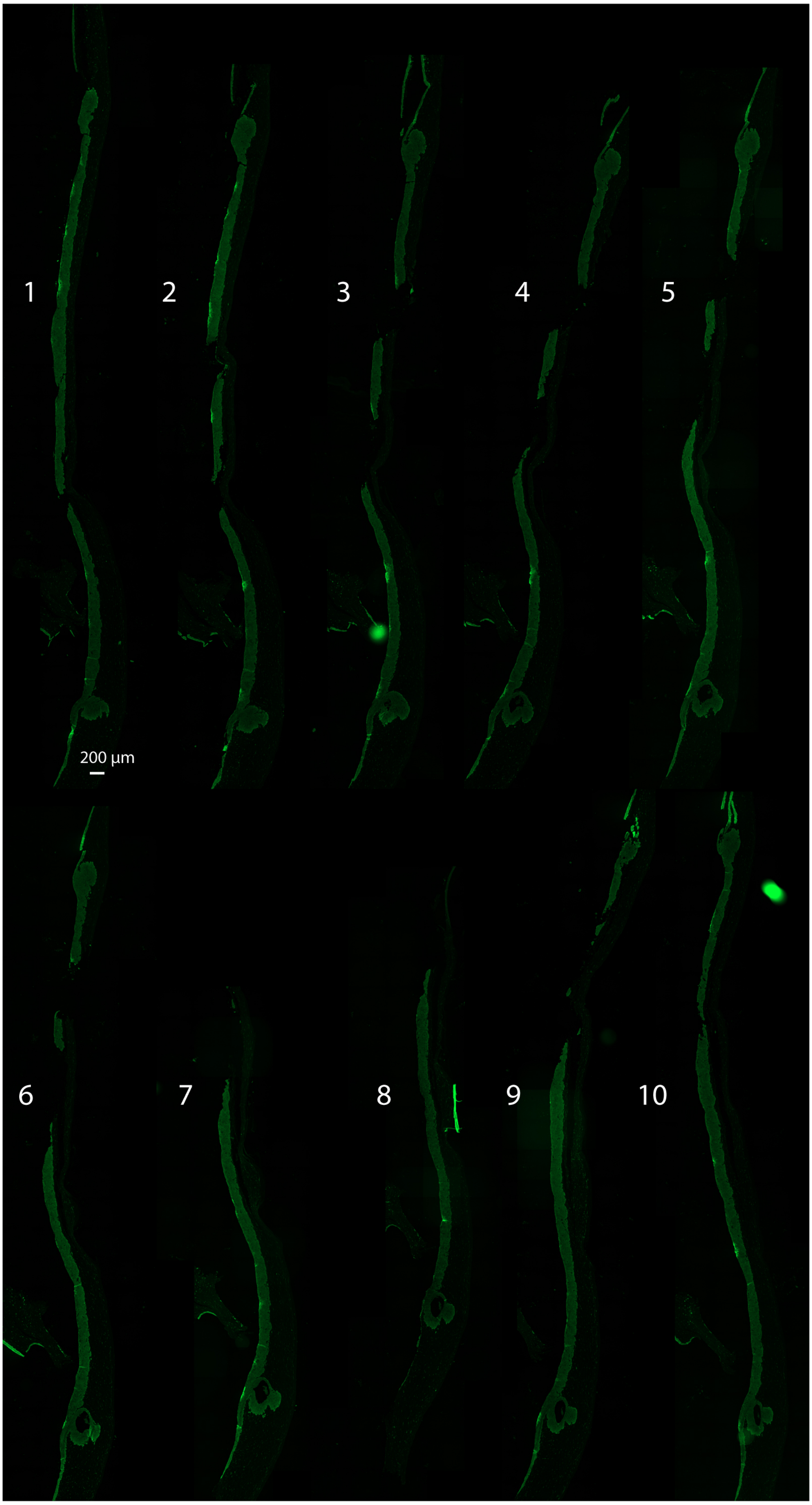

X01078Q *MGAT5* KO 1F12 + HSV-1 36 h section series 1

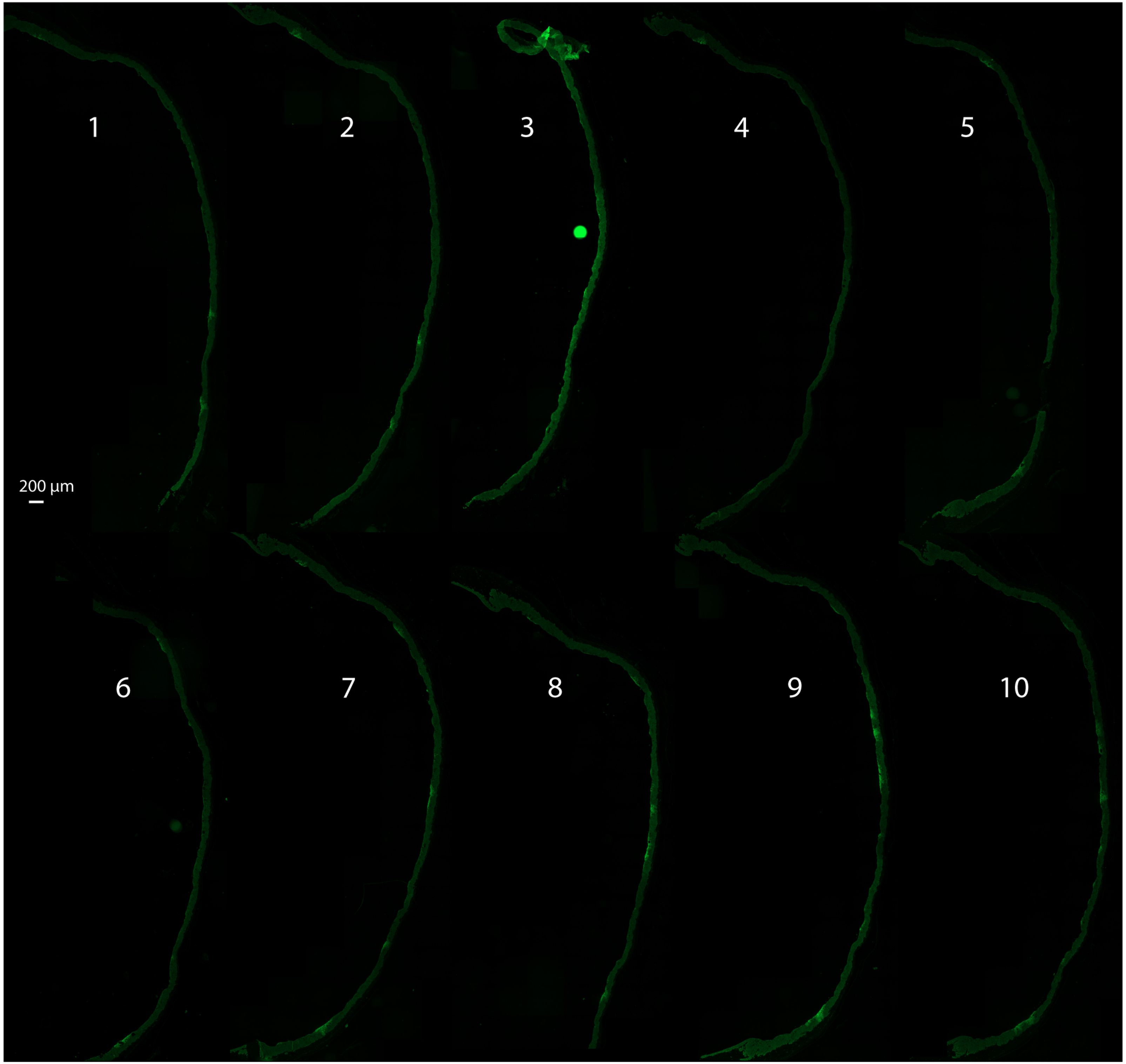

X01078Q *MGAT5* KO 1F12 + HSV-1 36 h section series 2

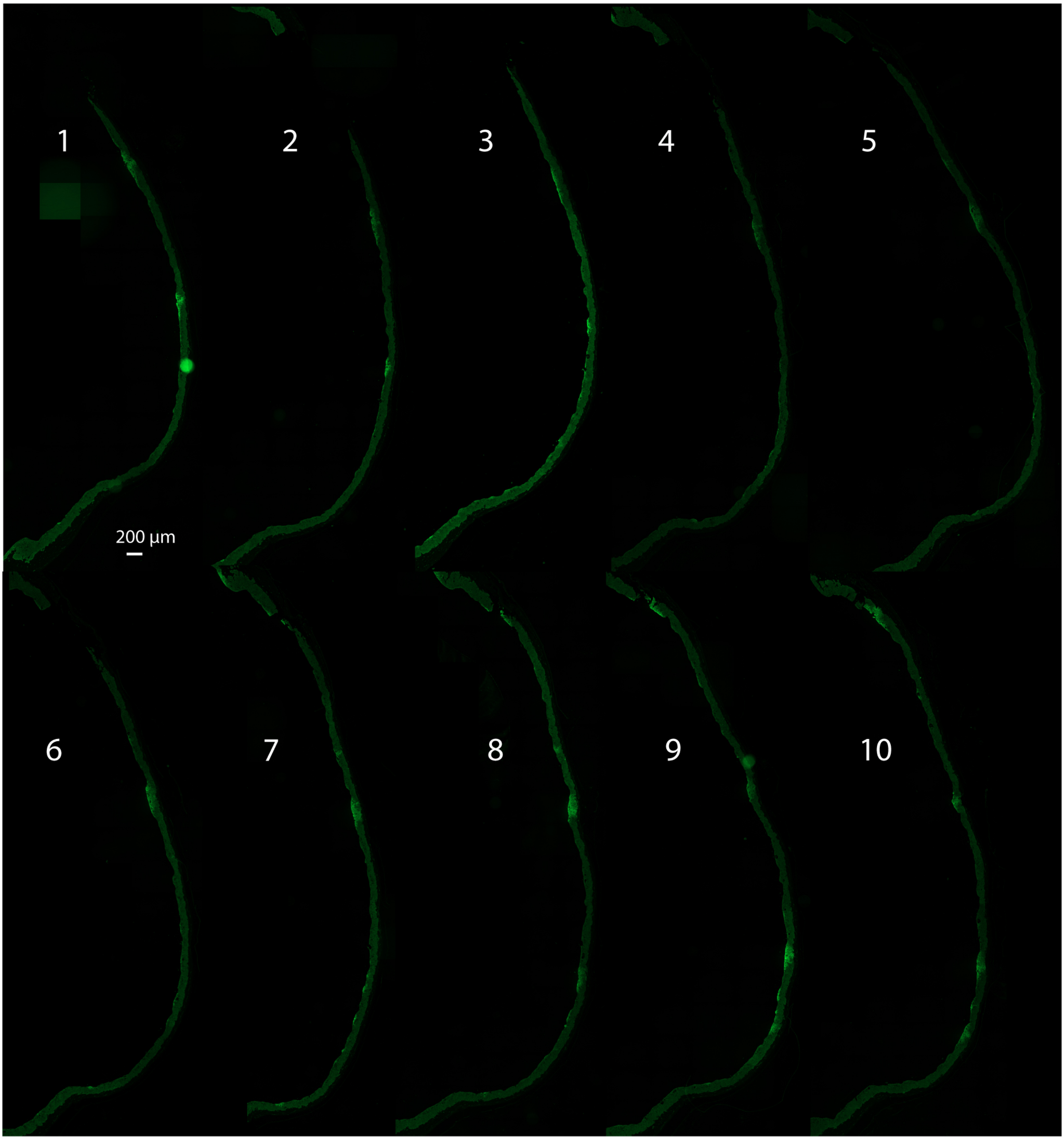

X01105R *MGAT5+4B* KO 2B5 + HSV-1 36 h section series 1

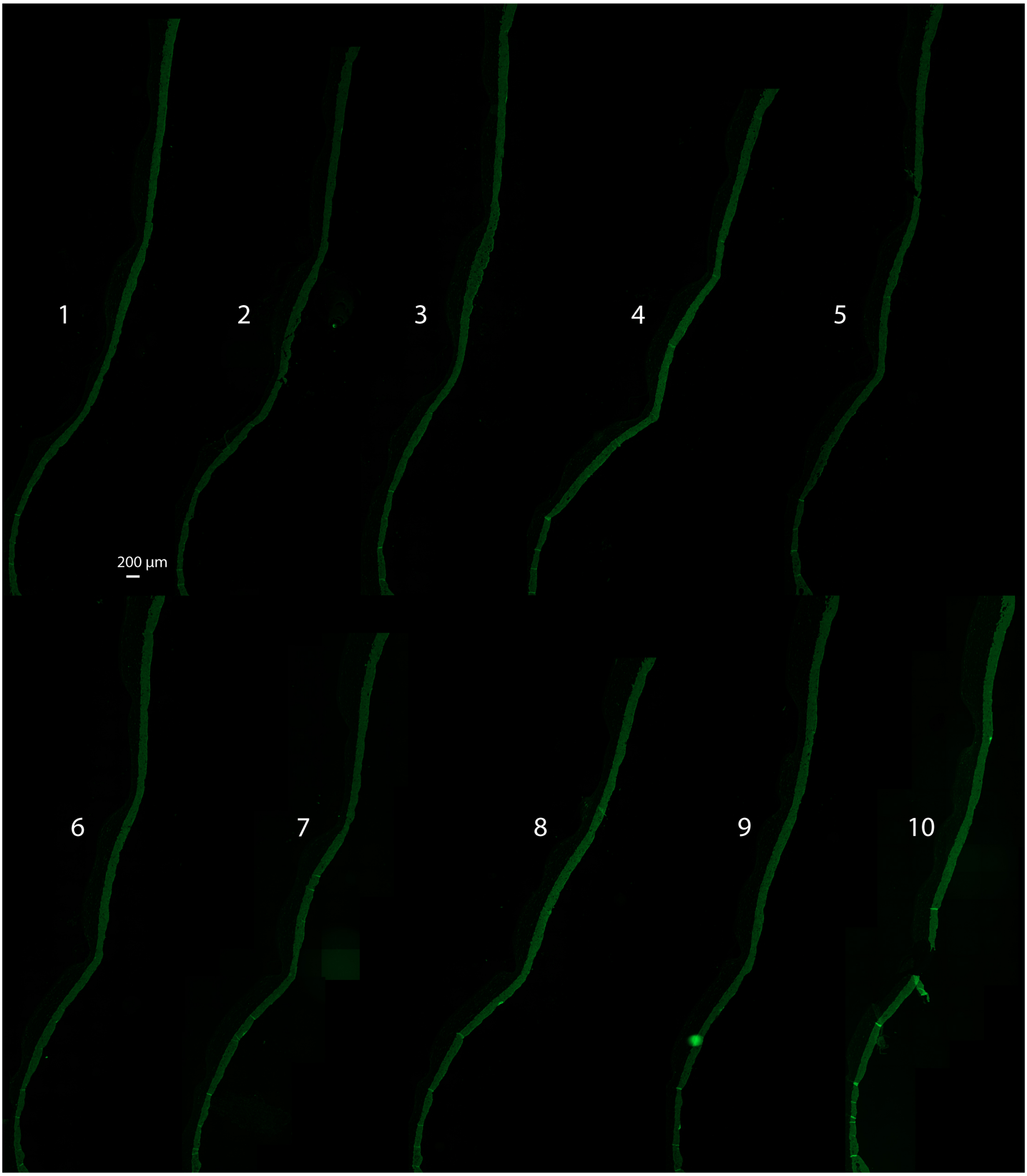

X01105R *MGAT5+4B* KO 2B5 + HSV-1 36 h section series 2

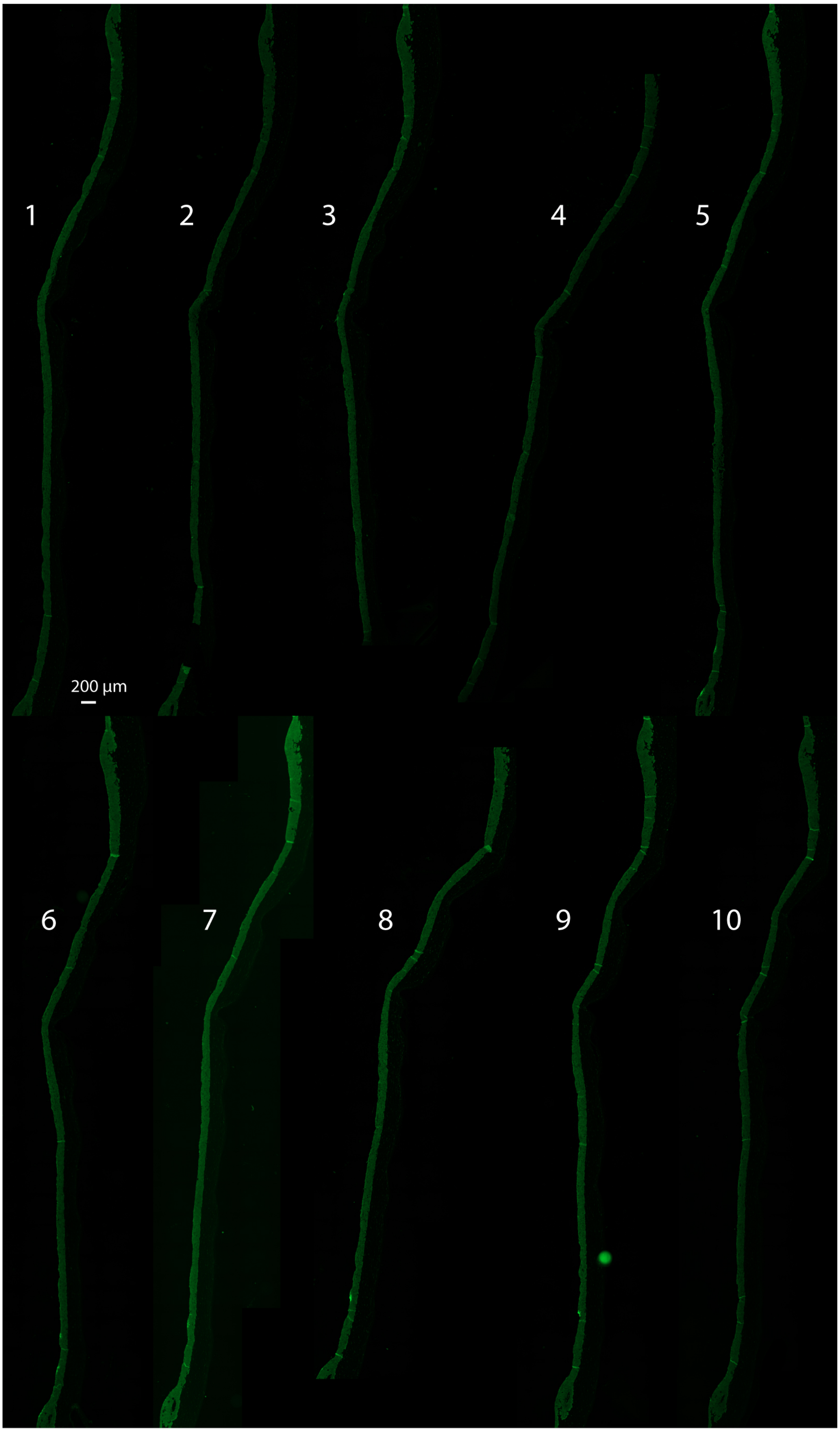

X01078N *C1GALT1C1* KO D5 + HSV-1 36 h section series 1

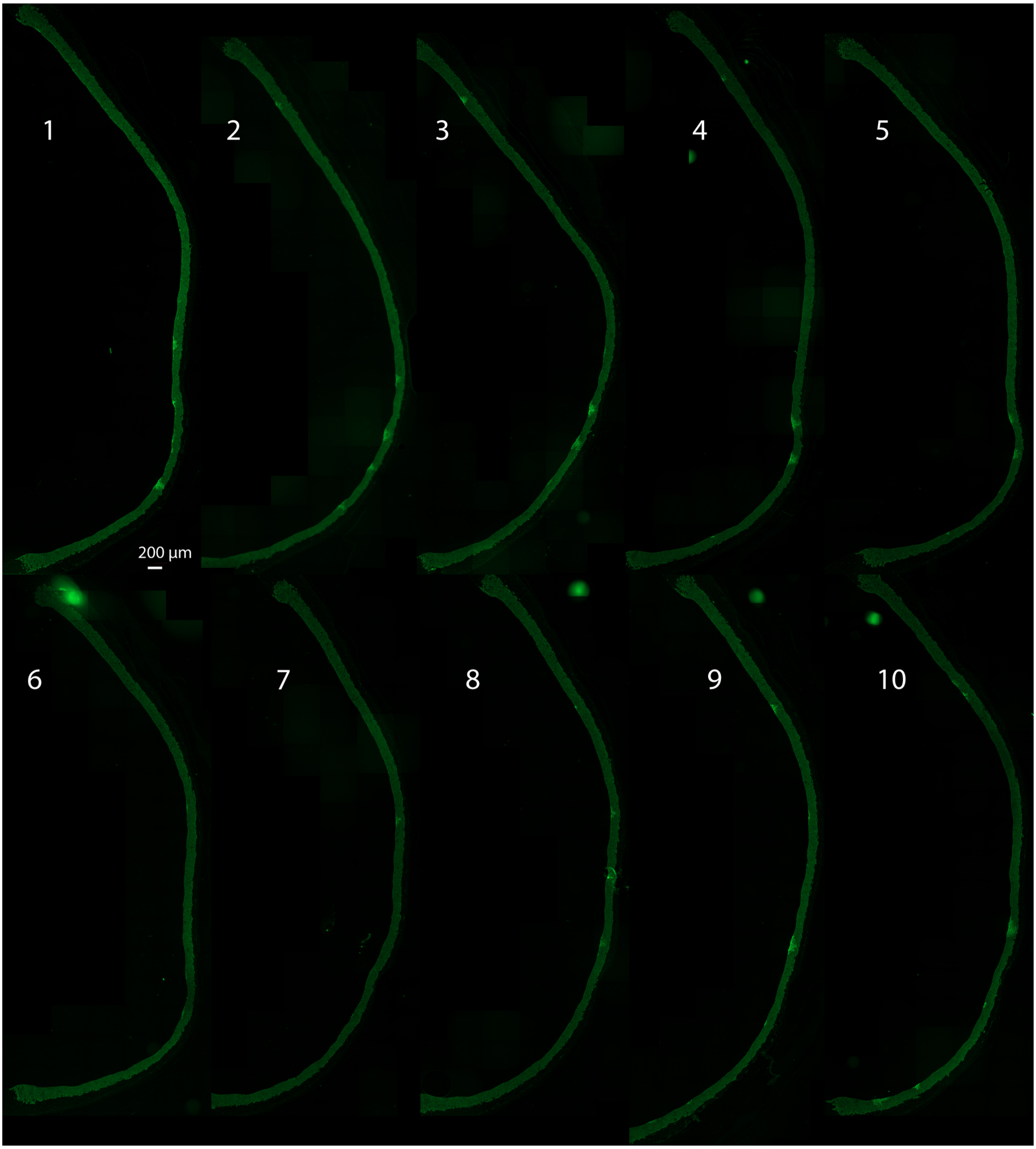

X01078N *C1GALT1C1* KO D5 + HSV-1 36 h section series 2

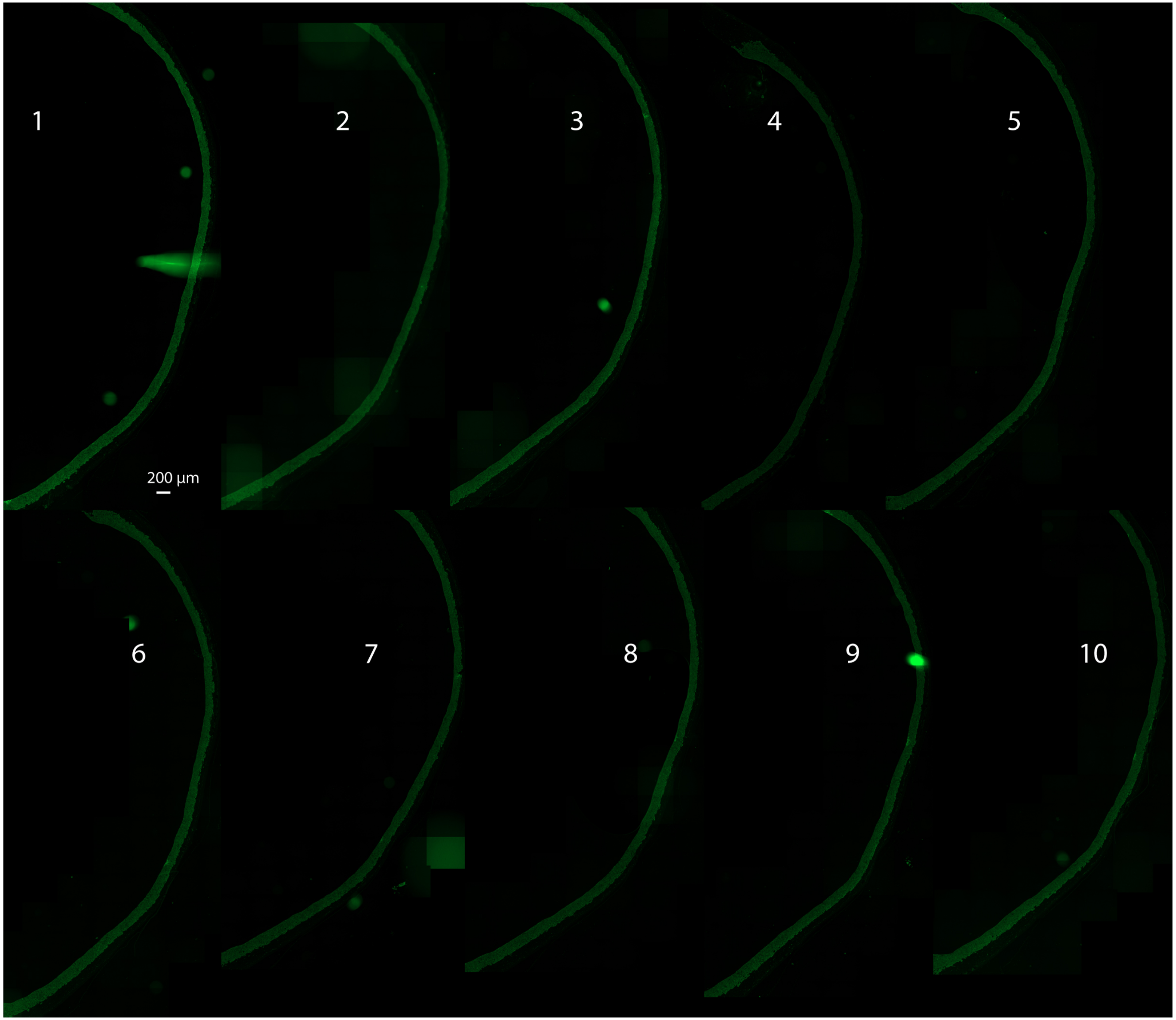

X01105I *C1GALT1* KO 2E5 + HSV-1 36 h section series 1

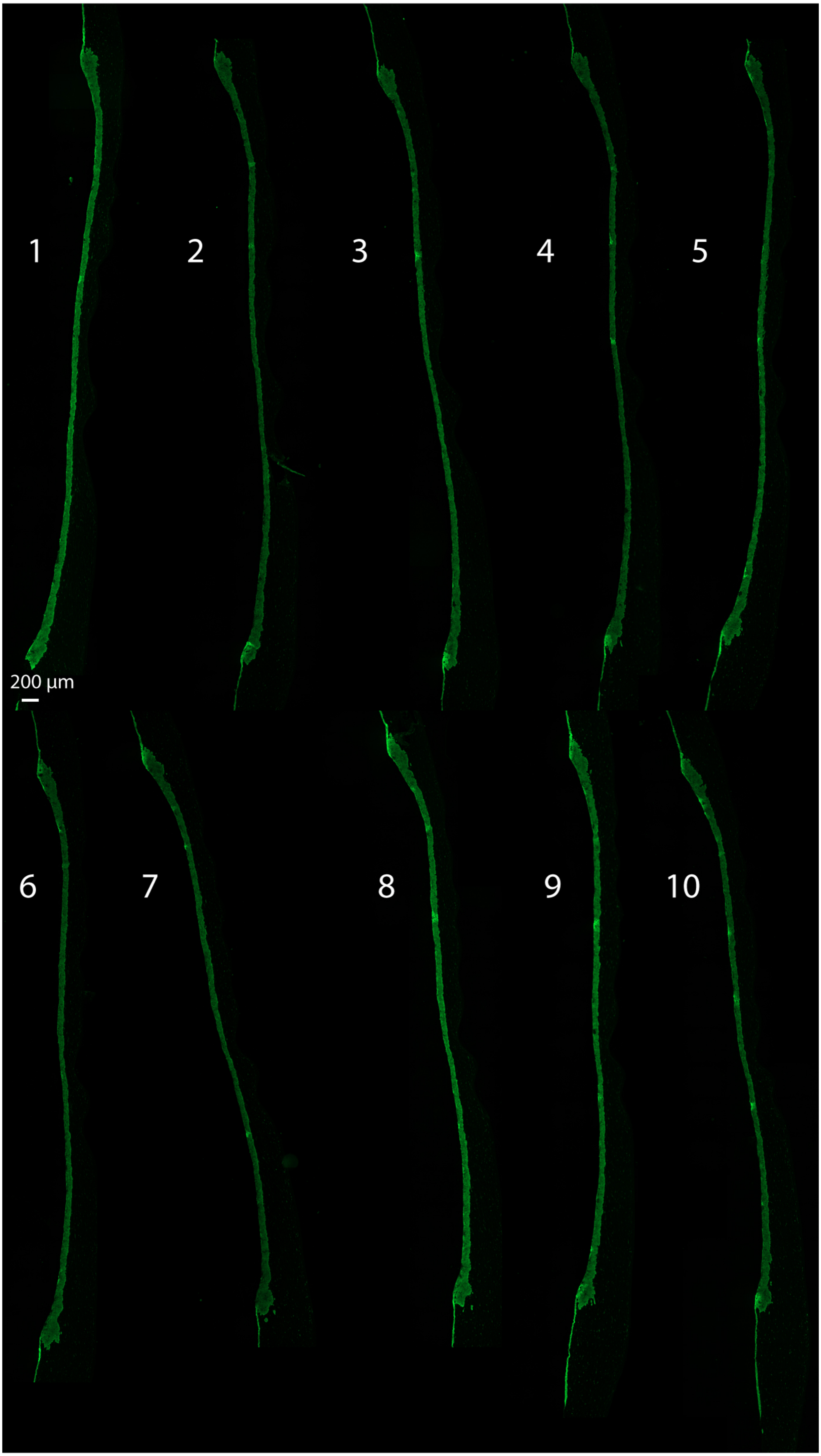

X01105I *C1GALT1* KO 2E5 + HSV-1 36 h section series 2

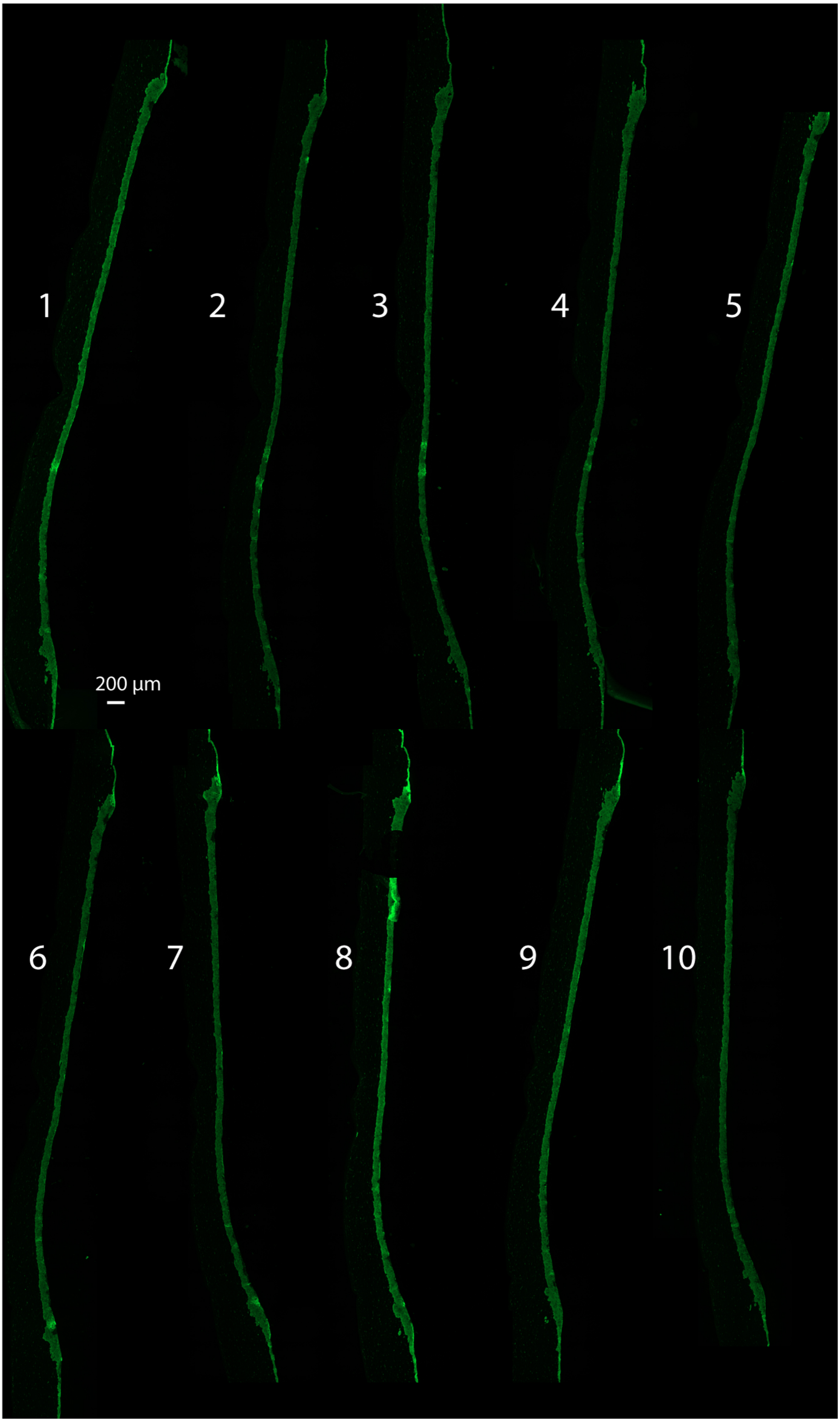

X01105Q *GCNT1* KO 2E7 + HSV-1 36 h section series 1

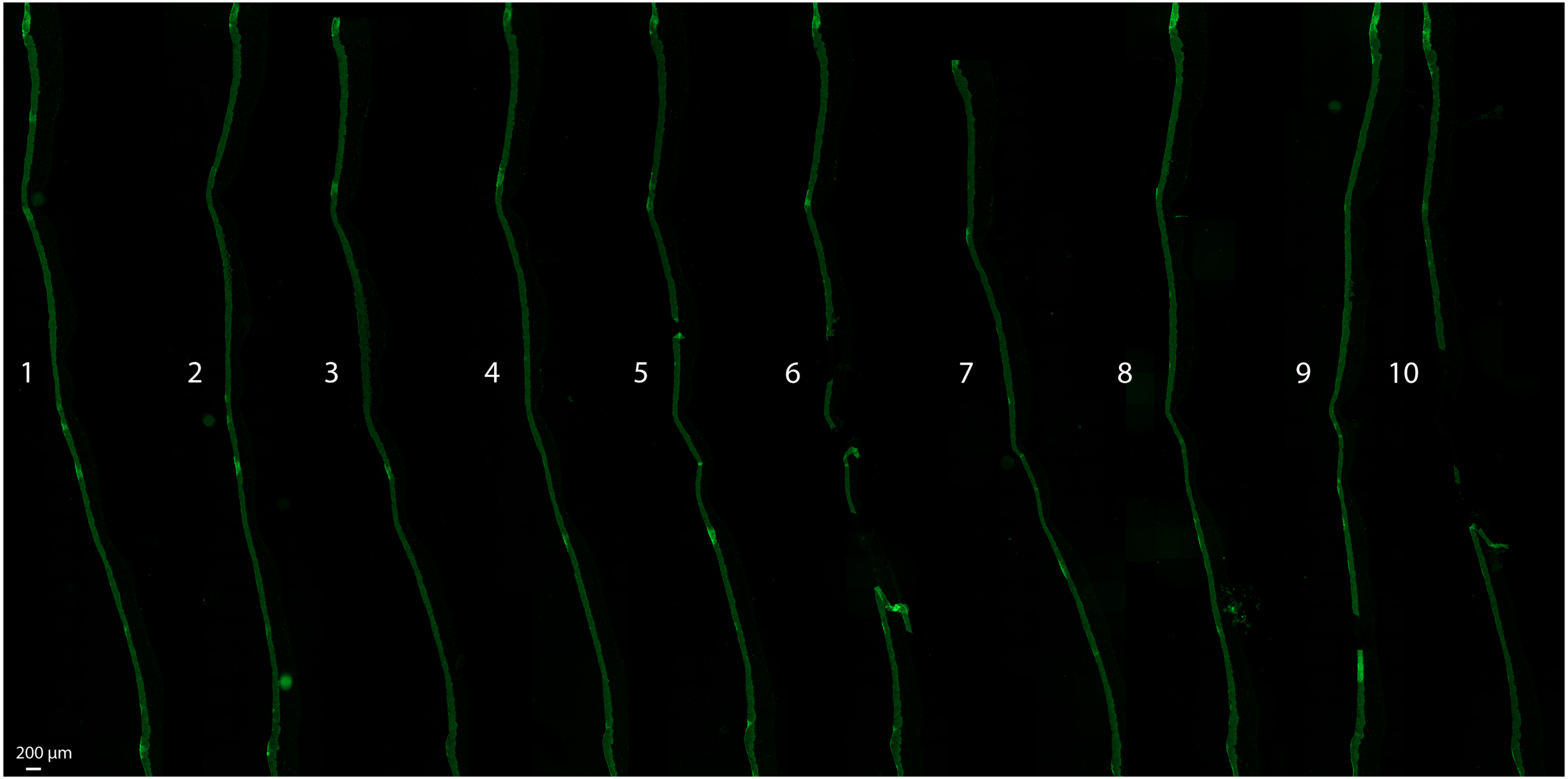

X01105Q *GCNT1* KO 2E7 + HSV-1 36 h section series 2

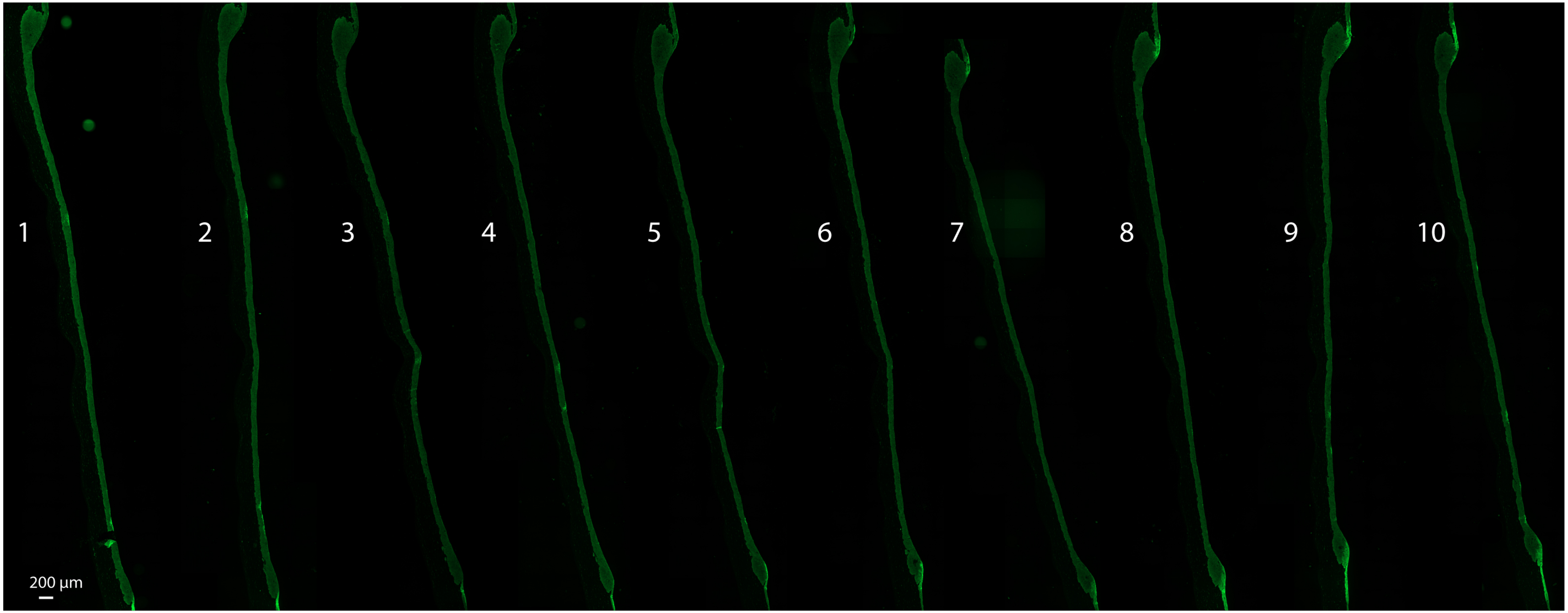

X01105K *ST3GAL1* KO 2B2 + HSV-1 36 h section series 1

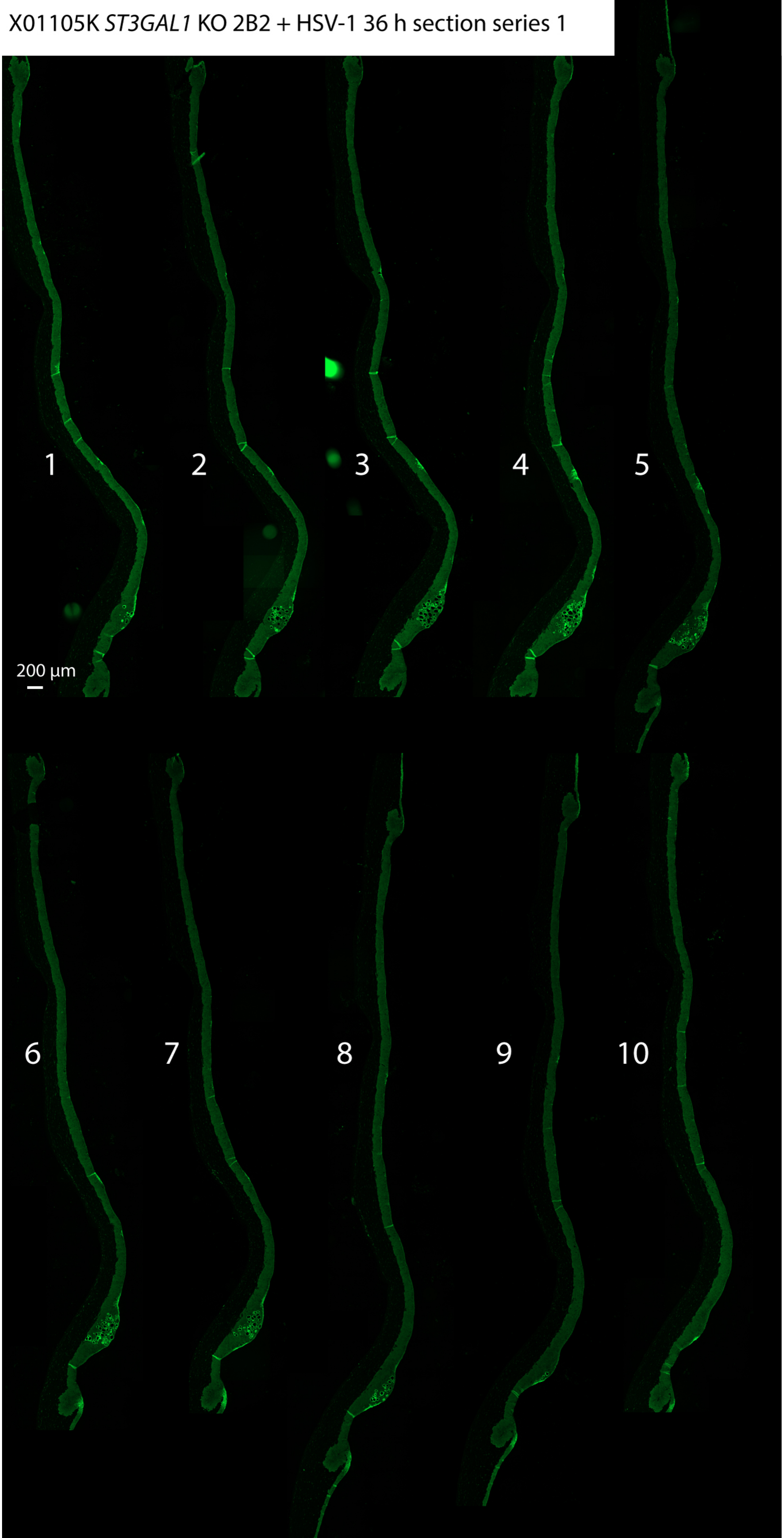

X01105K *ST3GAL1* KO 2B2 + HSV-1 36 h section series 2

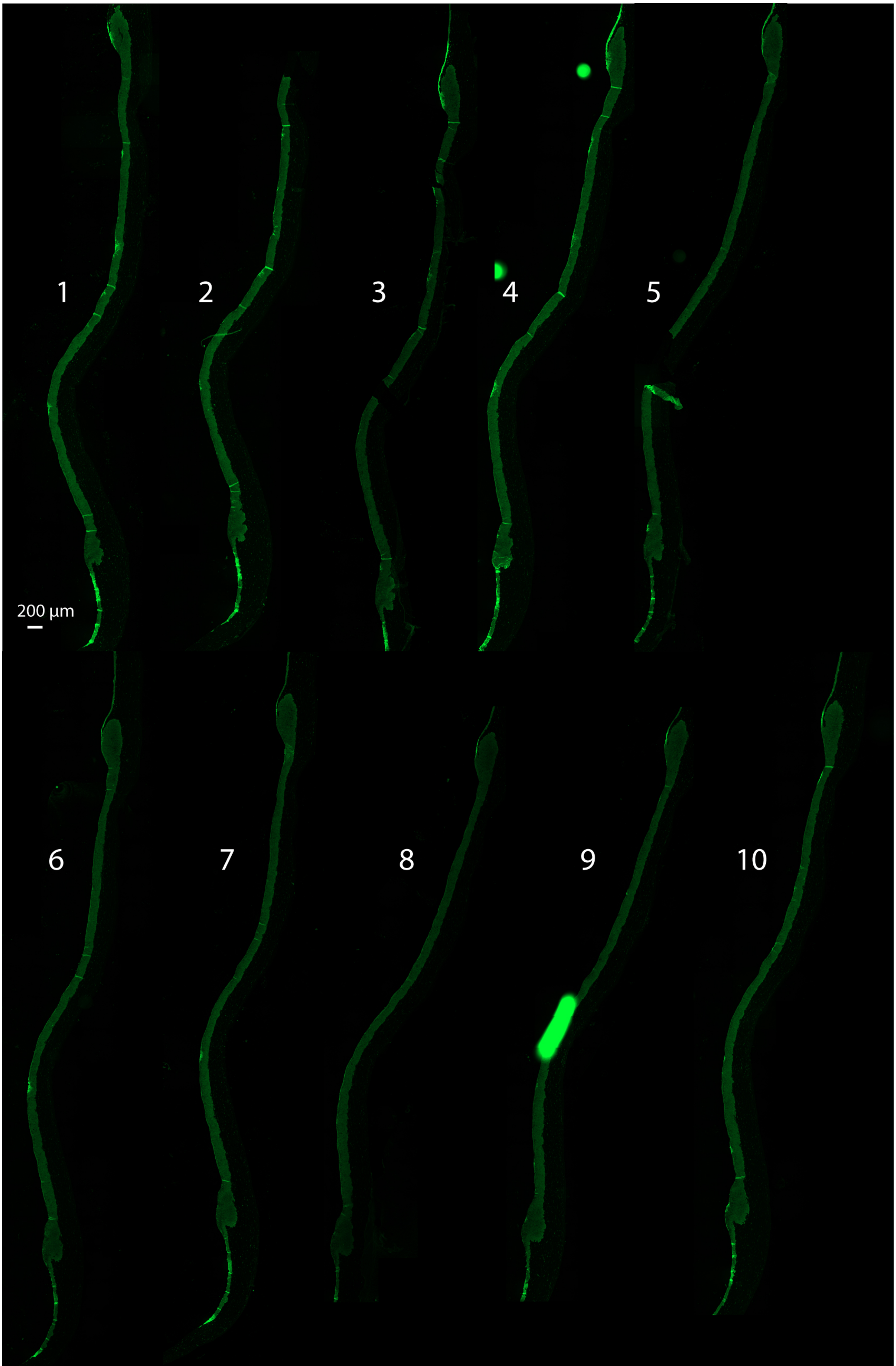

X01105H *B4GALT7* KO 3D9 + HSV-1 36 h section series 1

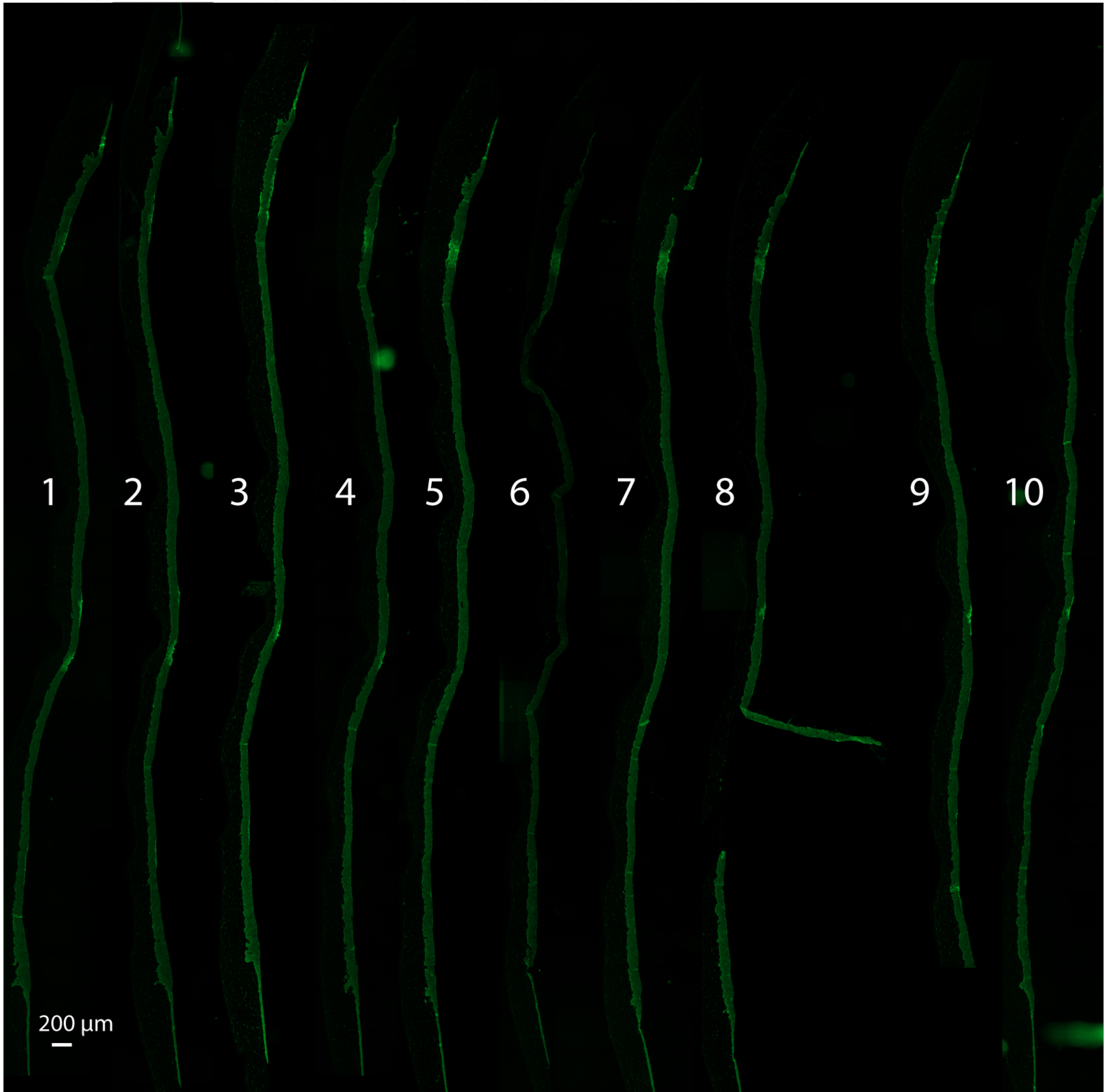

X01105H *B4GALT7* KO 3D9 + HSV-1 36 h section series 2

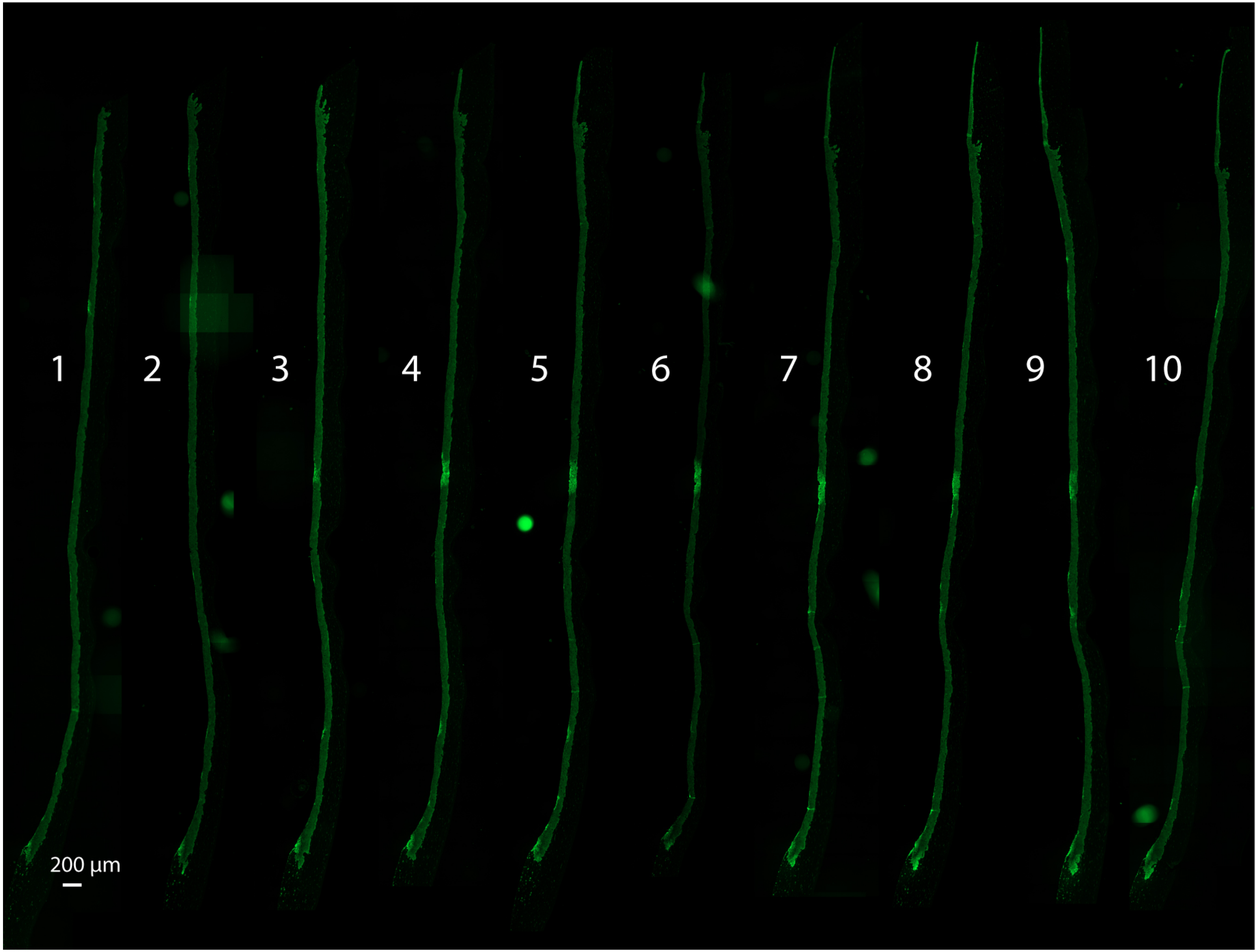

X01105J *B4GALT5* KO D11 + HSV-1 36 h section series 1

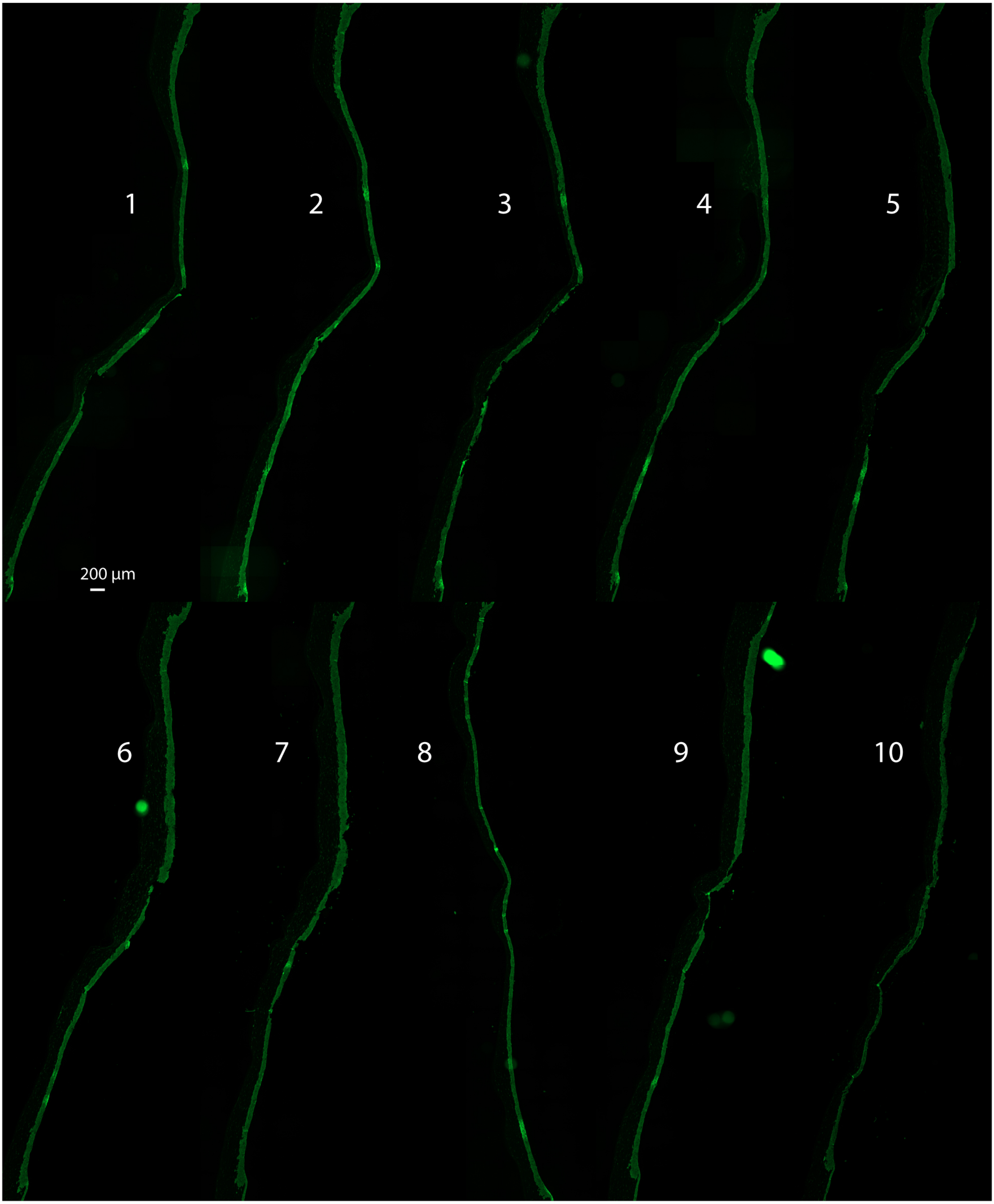

X01105J *B4GALT5* KO D11 + HSV-1 36 h section series 2

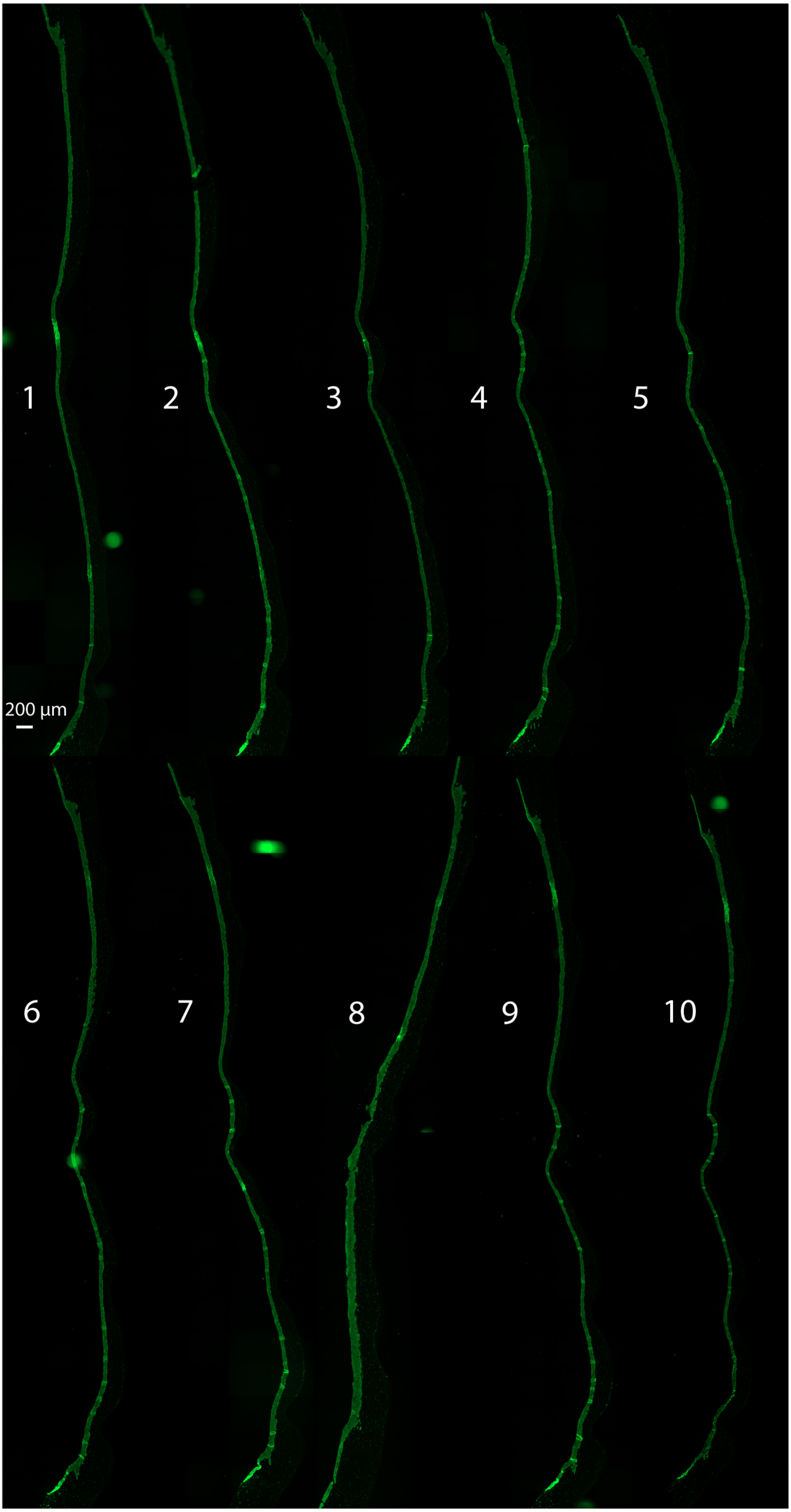

X01105P *ST3GAL5* KO 1C5 + HSV-1 36 h section series 1

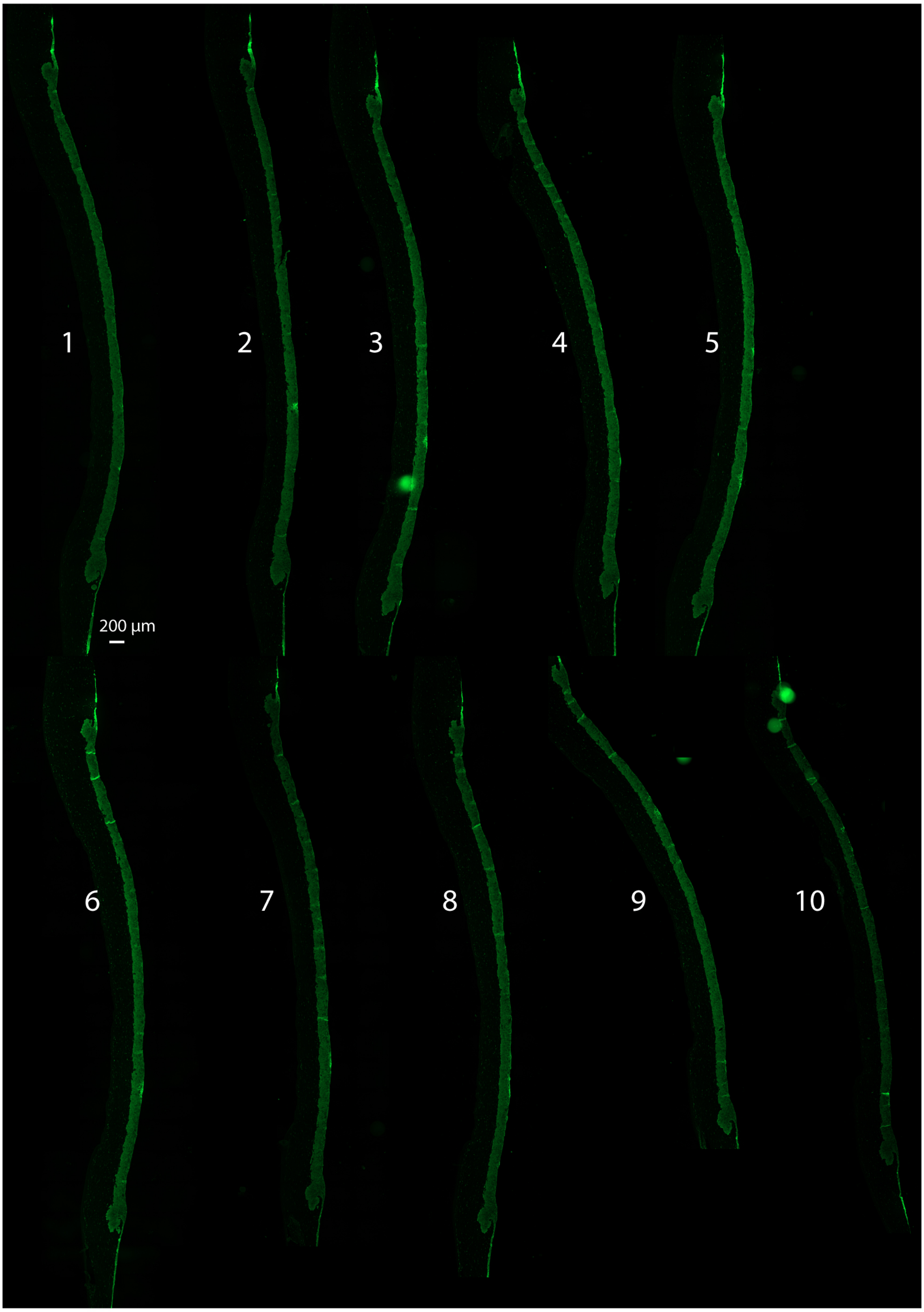

X01105P *ST3GAL5* KO 1C5 + HSV-1 36 h section series 2

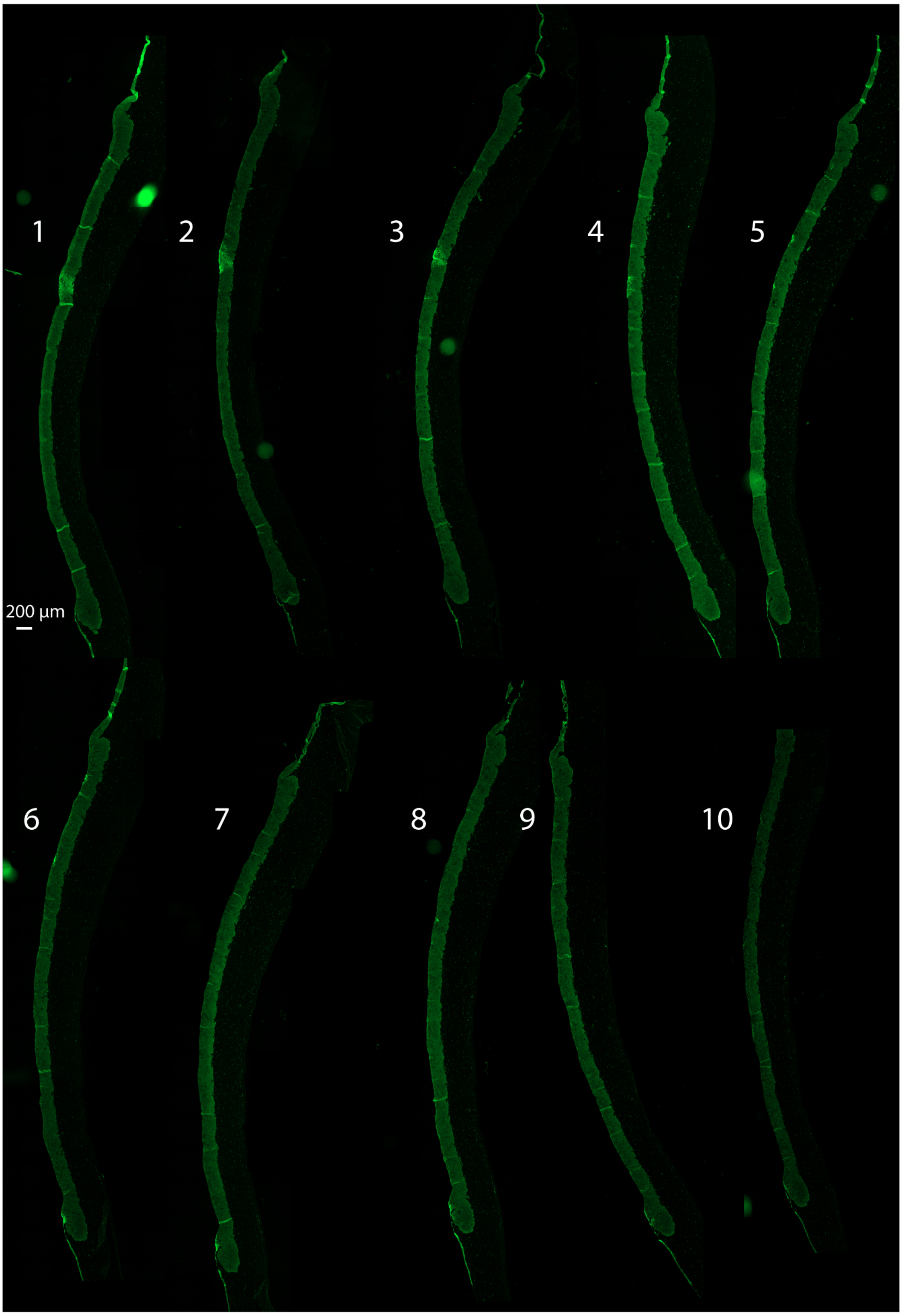

Supplement: Supplementary file 1 — Supplementary Information [file 41467_2023_42669_MOESM1_ESM.pdf]
