# Supplementary material for: Glycoengineered keratinocyte library reveals essential functions of specific glycans for all stages of HSV-1 infection
Source: Nat Commun. 2023 Nov 2;14:7000. doi: 10.1038/s41467-023-42669-6 (PMC10622544; doi:10.1038/s41467-023-42669-6)
Supplement: Supplementary file 2 — Reporting Summary [file 41467_2023_42669_MOESM2_ESM.pdf]

nature portfolio

Corresponding author(s): Hans H. Wandall, Ieva Bagdonaite

Last updated by author(s): Sep 21, 2023

Reporting Summary

Nature Portfolio wishes to improve the reproducibility of the work that we publish. This form provides structure for consistency and transparency in reporting. For further information on Nature Portfolio policies, see our Editorial Policies and the Editorial Policy Checklist.

Statistics

For all statistical analyses, confirm that the following items are present in the figure legend, table legend, main text, or Methods section.

n/a	Confirmed	
[ ]	[x]	The exact sample size (*n*) for each experimental group/condition, given as a discrete number and unit of measurement
[x]	[ ]	A statement on whether measurements were taken from distinct samples or whether the same sample was measured repeatedly
[ ]	[x]	The statistical test(s) used AND whether they are one- or two-sided<br>*Only common tests should be described solely by name; describe more complex techniques in the Methods section.*
[x]	[ ]	A description of all covariates tested
[ ]	[x]	A description of any assumptions or corrections, such as tests of normality and adjustment for multiple comparisons
[ ]	[x]	A full description of the statistical parameters including central tendency (e.g. means) or other basic estimates (e.g. regression coefficient) AND variation (e.g. standard deviation) or associated estimates of uncertainty (e.g. confidence intervals)
[ ]	[x]	For null hypothesis testing, the test statistic (e.g. *F*, *t*, *r*) with confidence intervals, effect sizes, degrees of freedom and *P* value noted<br>*Give P values as exact values whenever suitable.*
[x]	[ ]	For Bayesian analysis, information on the choice of priors and Markov chain Monte Carlo settings
[x]	[ ]	For hierarchical and complex designs, identification of the appropriate level for tests and full reporting of outcomes
[x]	[ ]	Estimates of effect sizes (e.g. Cohen's *d*, Pearson's *r*), indicating how they were calculated

Our web collection on statistics for biologists contains articles on many of the points above.

Software and code

Policy information about availability of computer code

Data collection	Zen Black 2012 SP5 (Zeiss) was used to document confocal images.
Data analysis	Zen Lite version 3.4.91.00000 (Zeiss), Adobe Photoshop 2021 version 22.3.0, and Adobe Illustrator 2021 version 25.2.1 were used to process images and assemble illustrations. PyMOL software version 2.1 was used to generate protein structure illustrations. Prism 9 software version 9.5.0 was used for data visualization and statistical analyses. FlowJo V. 10.0.7 software was used for flow cytometry data analysis.

For manuscripts utilizing custom algorithms or software that are central to the research but not yet described in published literature, software must be made available to editors and reviewers. We strongly encourage code deposition in a community repository (e.g. GitHub). See the Nature Portfolio guidelines for submitting code & software for further information.

Data

Policy information about availability of data

All manuscripts must include a data availability statement. This statement should provide the following information, where applicable:

- Accession codes, unique identifiers, or web links for publicly available datasets
- A description of any restrictions on data availability
- For clinical datasets or third party data, please ensure that the statement adheres to our policy

All data generated or analysed during this study are included in this published article (and its supplementary information files). Protein structure coordinate files (PDB: 2GUM (https://doi.org/10.2210/pdb2gum/pdb) and PDB: 2C36 (https://doi.org/10.2210/pdb2c36/pdb)) were downloaded from the Protein Data Bank for generating protein structure illustrations in PyMOL.

Human research participants

Policy information about studies involving human research participants and Sex and Gender in Research.

Reporting on sex and gender	n/a
Population characteristics	n/a
Recruitment	n/a
Ethics oversight	n/a

Note that full information on the approval of the study protocol must also be provided in the manuscript.

Field-specific reporting

Please select the one below that is the best fit for your research. If you are not sure, read the appropriate sections before making your selection.

- [x] Life sciences
- [ ] Behavioural & social sciences
- [ ] Ecological, evolutionary & environmental sciences

For a reference copy of the document with all sections, see nature.com/documents/nr-reporting-summary-flat.pdf

Life sciences study design

All studies must disclose on these points even when the disclosure is negative.

Sample size	Based on standard practice, 3 biological replicates of infected cells were used for viral replication experiments, each assayed in duplicate and triplicate by plaque assay and qPCR, respectively (doi: 10.1371/journal.ppat.1004784). 3 biological replicates were used for each binding or entry experiment (doi: 10.1128/JVI.01582-16). 2 technical replicates were used for flow cytometry and 3 replicates were used for kinetic measurements (cell-cell fusion assay, doi: 10.1016/j.ymeth.2015.05.021). Due to variability in viral plaque size, 50 plaques were measured for each different knock out cell line to collect a range of representative data.
Data exclusions	No data was excluded.
Replication	For each type of quantitative viral assay (titer, DNA copies, binding, entry), three independent experiments were performed for each cell line. For binding and entry assays the data is presented as WT-normalized means based on the three independent experiments. Flow cytometry data encompasses results from two or three independent experiments. For plaque size distribution, all measurements from three independent experiments were compiled and presented as violin diagrams. Kinetic measurements of cell-cell fusion activity are representative of two independent experiments, both shown in the graphs. All attempts at replication were successful for the above listed experiments. Imaging is representative of at least two independent experiments, as described in figure legends.
Randomization	Due to high labor-intensity, viral assays were performed using 3-4 cell lines at a time (including wild type in each experiment), grouped at a random order.
Blinding	Since individual experiments were set up and performed by specific people, it was impractical for us to assign other personnel to implement blinding in the large and time-sensitive experimental setups. Thus, no blinding was performed.

Reporting for specific materials, systems and methods

We require information from authors about some types of materials, experimental systems and methods used in many studies. Here, indicate whether each material, system or method listed is relevant to your study. If you are not sure if a list item applies to your research, read the appropriate section before selecting a response.

Materials & experimental systems

n/a	Involved in the study
☐	☒ Antibodies
☐	☒ Eukaryotic cell lines
☒	☐ Palaeontology and archaeology
☒	☐ Animals and other organisms
☒	☐ Clinical data
☒	☐ Dual use research of concern

Methods

n/a	Involved in the study
☒	☐ ChIP-seq
☐	☒ Flow cytometry
☒	☐ MRI-based neuroimaging

Antibodies

Antibodies used	Non-commercial monoclonal antibodies: mouse anti-gB (clone HSV-1B11D8); mouse anti-gC (clone B1C1B4); mouse anti-gD (clone C4D5G2); mouse anti-gE (clone B1E6A5). Commercial antibodies: rabbit anti-E-cadherin mAb (clone 24E10) - Cell Signaling Technologies cat# 3195; goat anti-HSV-1-FITC pAb - Genetex cat# GTX40437; mouse anti-Nectin 1 mAb (clone CK8) - Thermo Fisher Scientific cat# 37-5900; rabbit anti-HVEM pAb - Thermo Fisher Scientific cat# PA5-29780; mouse anti-ceramide mAb - Glycobiotech cat# MAB_0010; rabbit anti-glucosylceramide antiserum - Glycobiotech cat# RAS_0010; mouse anti-GM3 (clone CGYJ074) mAb - Creative Biolabs cat# AGM-200YJ; goat anti-mouse IgG AF647 F(ab)2 - Thermo Fisher Scientific cat# A-21237; goat anti-mouse IgG AF594 (H+L) - Thermo Fisher Scientific cat# A-11032; goat anti-mouse IgG AF488 (H+L) - Thermo Fisher Scientific cat# A-11001; donkey anti-rabbit IgG AF546 (H+L) - Thermo Fisher Scientific cat# A10040; goat anti-mouse IgM AF546 (H chain) - Thermo Fisher Scientific cat# A21045, goat anti-rabbit IgG AF488 (H+L) - Thermo Fisher Scientific cat# A-11008.
Validation	Non-commercial monoclonal antibodies against HSV-1 glycoproteins were validated by ELISA using purified viral proteins as antigens (doi: 10.1016/0890-8508(92)90070-e). According to manufacturer, Rabbit anti-E-cadherin mAb (clone 24E10) was validated by flow cytometry using E-cad non-expressing cell line as negative control (https://www.cellsignal.com/products/primary-antibodies/e-cadherin-24e10-rabbit-mab/3195). Goat anti-HSV-1-FITC pAb (GTX40437) does not stain non-infected cells. We validated mouse anti-Nectin 1 and rabbit anti-HVEM antibodies on cell lines stably expressing the respective molecules and a negative cell line. Glycobiotech anti-glycosphingolipid antibodies (anti-ceramide cat# MAB_0010 and anti-glucosylceramide cat# RAS_0010) were validated by the manufacturer using dot blots coated with synthetic glycosphingolipids. We validated the mouse anti-GM3 (clone CGYJ074) mAb on our wild type and glycosphingolipid knock out skin cell lines lacking this structure.

Eukaryotic cell lines

Policy information about cell lines and Sex and Gender in Research

Cell line source(s)	All knock out cell lines were generated at Copenhagen Center for Glycomics, Department of Cellular and Molecular Medicine, University of Copenhagen, Denmark. The parental cell lines were either obtained from other laboratories or purchased. The HaCaT keratinocyte cell line (male) was originally generated by Petra Boukamp and Norbert Fusenig at the German Cancer Research Center, Heidelberg, Germany (Boucamp et al., J Cell Biol 1988 (PMID: 2450098 DOI: 10.1083/jcb.106.3.761)). HEK293 human cell line (female) was purchased from Sigma (cat. Nr. 85120602). CHOZN GS -/- (glutamine synthetase KO) (female) was also purchased from Sigma. Green monkey kidney (GMK) cell line was originally obtained from the Swedish Institute for Infectious Disease Control, Stockholm (Guenalp, Proc Soc Exp Biol Med 1965 (PMID: 14254593)). CHO-Nectin 1 (HveC), CHO-HVEM and the parental CHO cell line (female) were obtained from Richard Longnecker.
Authentication	Each engineered cell line was confirmed several times by gene specific IDAA (indel deletion by amplicon analysis) and Sanger sequencing in the target gene area.
Mycoplasma contamination	A representative set of engineered cell lines has been tested for Mycoplasma contamination during routine screening procedures, testing negative.
Commonly misidentified lines (See ICLAC register)	No commonly misidentified cell lines were used in the study.

Flow Cytometry

Plots

Confirm that:

☒ The axis labels state the marker and fluorochrome used (e.g. CD4-FITC).

☒ The axis scales are clearly visible. Include numbers along axes only for bottom left plot of group (a 'group' is an analysis of identical markers).

☒ All plots are contour plots with outliers or pseudocolor plots.

☒ A numerical value for number of cells or percentage (with statistics) is provided.

Methodology

Sample preparation	For the analysis of suspension CHO cell lines, the cells were counted and 1E+05 cells/well were distributed into U-bottom 96-

well plate (TermoFisher Scientific Nunclon Delta surface). Cell viability was evaluated by trypan blue stain, applying a viability requirement of > 95 % for cells to be included for analysis. Next, cells were washed once in ice-cold PBS, centrifuged at 500 x g for 3 min at 4 °C. Cells were then incubated with recombinant gC protein (0.5 µg/mL) diluted in ice-cold PBS with 2 % FBS, for 45 min at 4 °C. Following, cells were washed twice in ice-cold PBS with 2 % FBS before incubation with mouse anti-gC mAb B1C1B4 (2 µg/mL) diluted in PBS with 2 % FBS for 45 min at 4 °C. After incubation, cells were washed twice in ice-cold PBS with 2 % FBS and incubated with goat anti-mouse IgG (H+L) AF488 (1:1000) diluted in PBS with 2 % FBS for 45 min at 4 °C in the dark. Lastly, cells were washed twice with PBS with 2 % FBS, and suspended in 100 µL PBS with 2 % FBS for analysis. For the analysis of HaCaT, CHO K1, CHO-Nectin 1 (HveC), CHO-HVEM, and HEK293 cells, 4E+05 cells/well were used. In addition, a cell viability stain (eBioscience™ Fixable Viability Dye eFluor™ 450, 1:1000 in PBS) for 30 min at 4 °C prior to antibody labelling was included. The cells were then incubated with relevant primary (mouse anti-Nectin 1 or rabbit anti-HVEM 1:50 in 1 % BSA in PBS) and subsequent secondary (goat anti-mouse IgG AF488 (H+L) or goat anti-rabbit IgG AF488 (H+L) 1:1000 in 1 % BSA in PBS) Abs for 30 min at 4 °C each. Cells were washed with 1 % BSA in PBS thrice between all the incubation steps. Incubation with secondary antibodies only were used for background subtraction.

Instrument

SONY Spectral Cell Analyzer (SONY SA3800) was used for data collection.

Software

All data analysis was done using FlowJo V. 10.0.7.

Cell population abundance

Not applicable - gating was only performed to exclude cell debris and doublets.

Gating strategy

A total minimum of 10 000 events were recorded for analysis. Cell debris and doublets were excluded based on FSC-A/FSC-H or SSC-A/SSC-H plots to ensure analysis based on singlets.

☒ Tick this box to confirm that a figure exemplifying the gating strategy is provided in the Supplementary Information.
